# A Novel Drug-Mouse Phenotypic Similarity Method Detects Molecular Determinants of Drug Effects

**DOI:** 10.1371/journal.pcbi.1005111

**Published:** 2016-09-27

**Authors:** Jeanette Prinz, Ingo Vogt, Gianluca Adornetto, Mónica Campillos

**Affiliations:** 1 Institute of Bioinformatics and Systems Biology, Helmholtz Zentrum München, Neuherberg, Germany; 2 German Center for Diabetes Research, Helmholtz Zentrum München, Neuherberg, Germany; Tufts University, UNITED STATES

## Abstract

The molecular mechanisms that translate drug treatment into beneficial and unwanted effects are largely unknown. We present here a novel approach to detect gene-drug and gene-side effect associations based on the phenotypic similarity of drugs and single gene perturbations in mice that account for the polypharmacological property of drugs. We scored the phenotypic similarity of human side effect profiles of 1,667 small molecules and biologicals to profiles of phenotypic traits of 5,384 mouse genes. The benchmarking with known relationships revealed a strong enrichment of physical and indirect drug-target connections, causative drug target-side effect links as well as gene-drug links involved in pharmacogenetic associations among phenotypically similar gene-drug pairs. The validation by *in vitro* assays and the experimental verification of an unknown connection between *oxandrolone* and prokineticin receptor 2 reinforces the ability of this method to provide new molecular insights underlying drug treatment. Thus, this approach may aid in the proposal of novel and personalized treatments.

## Author Summary

In order to avoid unwanted effects of current drug interventions, it is necessary to expand the knowledge of the molecular mechanisms related to drug action. Side effects offer insight into drug action, as for example similar side effects of unrelated drugs can be caused by their common off-targets. Moreover, the phenotypes of systematic single gene perturbation screenings in mice strongly contribute to the comprehension of gene function. Here, we present a novel approach that detects molecular interactions of drugs based on the phenotypic similarity of drugs and mouse models. The method is benchmarked with diverse data sets including drug-target interactions as well as gene-drug links of pharmacogenetic associations and validated by *in vitro* assays.

## Introduction

A drug can modulate its targets directly or indirectly (e.g. via modulation of the gene expression) and only a small proportion of these protein targets are known [1–3]. Due to this incomplete understanding of drug mode of action, current drug treatment often suffers from unwanted effects [4]. In addition, the promiscuity of many drugs, that is the tendency of drugs to modulate multiple targets [5], hampers the anticipation of drug response and adverse effects in clinical practice. This is furthermore complicated by the genomic heterogeneity in the population, which produces a large variability of efficacy and adverse effects among patients [6]. Pharmacogenomic studies fortified the important role of gene sequence polymorphisms in drug efficacy and adverse effects [7–9]. Understanding each individual’s drug response is, thus, an additional challenge in the treatment of diseases and has a huge impact on attrition rates in drug discovery. Therefore, in order to personalize medication and to improve drug efficacy as well as drug safety, it is necessary to develop novel approaches expanding the knowledge of the molecular mechanisms underlying drug treatment.

Several experimental techniques have been developed to detect molecular associations of drugs [10]. However, limitations on the identifiable drug targets and their indirect effects, the high cost and low throughput of those experiments have hindered the elucidation of molecular determinants of many drugs. Classical approaches to detect drug-target interactions are based on biochemical affinity purification [11]. This method is time consuming and can only detect abundant high-affinity binding proteins, hampering its applicability to detect indirect and low affinity associations as well as interactions with protein complexes. Chemical proteomics approaches that typically combine affinity chromatography and proteomic techniques [12] have the advantage of finding interactions on a large scale. Yet, the challenge persists to detect interactions with proteins expressed at low levels without including unspecific bindings. Expression-cloning-based methods, like phage display or yeast three-hybrid [13], can circumvent the low protein abundance issue [14], but they cannot always capture the complexity of molecular and chemical interactions in the human organism [15].

Computational methods are arising as alternative and complementary approaches to propose novel molecular drug interactions. Methods relying e.g. on structural similarity of compounds [4, 16] or side effect similarity have been successfully applied to reveal drug-target relationships and also to provide mechanistic insights into adverse effects [5, 17, 18]. Recently, the comparison of side effects of drugs and phenotypic traits of perturbed genes in mouse models has also been proposed as an option to identify drug targets [19]. Interestingly, this approach has the advantage of not relying on established drug-target relationships, offering the potential to discover novel drug-target interactions. This method follows the idea that the manipulation of a target by genetic or pharmacological means should consistently lead to phenotypic changes that are aligned with the desired therapeutic effect [20]. In this aspect, it has been shown that phenotypes resulting from knock-out mice correlate well with known phenotypes of drug response [21]. However, to detect single gene perturbations in mice that share similar phenotypes with drugs in a sensitive manner, several methodological challenges need to be overcome. These challenges arise from the large number of side effects of drugs stemming from their polypharmacological potential [22, 23] as well as physiological differences between mice and humans.

In this work, we develop a new scoring scheme to evaluate the similarity of phenotypic traits from gene perturbations in mice and side effect profiles of drugs, which is able to cope with the polypharmacological property of drugs. Our approach reveals molecular associations of drugs and genes including direct and indirect drug targets, gene-drug links involved in pharmacogenetic associations as well as causal protein-side effect relationships. We moreover provide experimental evidence of the capability of our phenotypic similarity scoring scheme to detect novel drug-target interactions.

## Results

### Phenotypic similarity of drugs and genes

In order to learn more about drug mode of action and molecular mechanisms underlying side effects, we devised an extended semantic similarity scoring system to identify drugs and mouse genes that share similar phenotypes and are, thus, likely to be molecularly related [24]. As we aimed to find perturbations of mouse genes reproducing the side effects of drugs in human, we encoded the phenotypic traits in mice and the drug side effects with the Medical Dictionary for Regulatory Activities (MedDRA) [25]. MedDRA is a highly standardised medical terminology used for the annotation of adverse side effects in clinical trials that contains terms related to human health. Besides, an adaptation of the hierarchical organization of MedDRA (see Materials and Methods for details) enables the assessment of the semantic distance between phenotypes of different perturbations in mammalian organisms [26].

To determine drug—mouse gene pairs with similar phenotypes, we conceived a symmetric score that averages the drug-gene and gene-drug phenotypic similarities (see Materials and Methods for details) and devised an approach accounting for the well-known tendency of drugs to bind multiple targets [4], also known as polypharmacological property.

**Fig 1:**
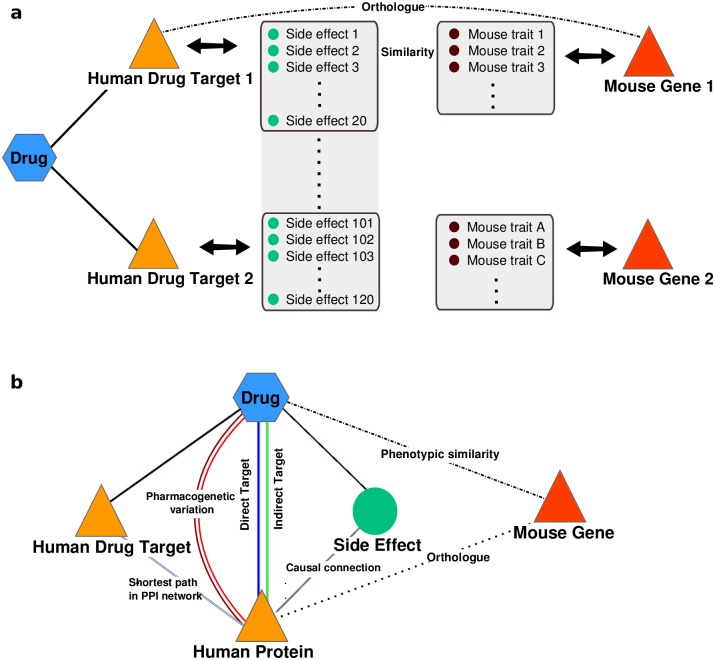
Schematic representation of the phenotypic similarity approach and validation of the method. a) Illustration of the phenotypic similarity approach that corrects for the polypharmacological property of drugs. A drug may affect multiple targets leading to more side effects than the one resulting from single gene perturbations in mice [23]. We therefore utilized only a subset of 20 side effects (see S2 and S3 Figs for cutoff evaluation) that are most similar to the mouse phenotypic traits to assess the similarity between a mouse gene and a drug. b) We evaluated our method with benchmark sets of direct and indirect human drug-target relationships, functional associations mediated through drug targets via protein-protein interactions, gene-drug pairs involved in pharmacogenetic associations and causal connections between human genes and side effects.

To compute the gene-drug phenotypic similarity, we averaged the scores of the most similar side effect-trait pair for all gene phenotypic traits, thereby prioritizing drugs that share a large proportion of their side effects with traits of mouse models. In contrast, for the computation of the drug-gene phenotypic similarity, we averaged only a subset of the 20 side effects with the highest phenotypic trait-side effect scores (see S2 and S3 Figs for cut-off evaluation). In this way, we disregarded side effects likely to be unrelated to a single target, thereby correcting for the polypharmacology of drugs (Fig 1a). In addition, as associations of drugs with many side effects to genes with low phenotypic information are more likely to score high by chance, we scaled the resulting score with the number of mouse phenotypic traits and downweighted drugs with many side effects (see Materials and Methods for details). This phenotypic similarity measurement allowed us to calculate the similarity of over 8 million gene-drug pairs involving 1,667 drugs and 5,834 single gene perturbations in mice.

In order to assess if our method can give insights into drug mode of action as well as associations between drug targets and adverse effects, we compared the phenotypic similarity of the gene-drug pairs to different data sets providing information of relationships between drugs, genes, and side effects (see Fig 1b). We tested if our approach detects known direct and indirect drug-target relationships, including those mediated through drug targets via protein-protein interactions, pharmacogenetic variations, and causal connections between proteins and side effects.

### Drug-target relationships

**Fig 2:**
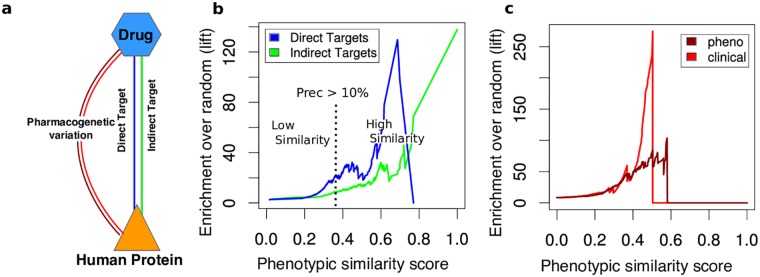
Benchmarking of the phenotypic similarity method. a) Overview over different benchmarking approaches used to validate the phenotypic similarity scoring scheme. b) Enrichment over random of direct (blue) and indirect (green) gene-drug associations benchmarked with drug-target associations from the STITCH database. The gene-drug pairs are classified as high (low) scoring if their phenotypic similarity is higher (lower) than the score at a precision of 10%. c) Enrichment over random of gene-drug associations of pharmacogenetic interactions from clinical (light red) and phenotypic annotations (dark red) in PharmGKB. The low lift values for very high scoring pairs (scores higher than 0.6) is explained by the sparse number of pairs with these scores within this benchmark set (see S7 Fig). Manual literature inspection of these pairs suggests that our method reveals genes involved in pharmacogenetic interactions also in the very high scoring regions.

First, we evaluated if our method is able to detect known drug-target associations from the STITCH database [27]. This dataset includes direct interactions, that is, physically interacting drug-target pairs (863,074 pairs) and drugs that modulate the targets indirectly (4,118,052 pairs), e.g. by altering the expression pattern of a gene via DNA binding (Fig 2a). We assessed the performance of our model using precision, ROC and lift measurements (Fig 2b and S3 Fig as well as S5 Fig). The lift value is a measurement of the performance of a method evaluated against a random choice model that estimates the precision of a scoring scheme in relation to the probability of obtaining a true value by chance (see Materials and Methods for details). We found that phenotypically related pairs are strongly enriched in both direct and indirect gene-drug molecular interactions (see Fig 2b). We also compared the performance of our model with a previously proposed gene-drug semantic similarity scoring system [24] and observed that our semantic similarity approach outperforms it significantly (see S4 Fig).

For a close manual inspection of the most confident gene-drug links detected by our approach, we focused on the set of high scoring gene-drug associations where the precision in the direct associations exceeds 10% and the lift reaches a value of 20 (Fig 2b) (1338 associations connecting 214 genes and 394 drugs). For indirect targets, we obtained an enrichment over random (lift value) of over 7 at this cutoff. We provide the full list of high scoring pairs in S1 Table. In addition, examples mentioned herein are shown in Table 1. Instances of direct connections among the high phenotypic similarity relationships include the antipsychotic drugs *aripiprazole* and *risperidone* linked to their direct target dopamine receptor 2 (*DRD2*), the Vitamin D Receptor (*VDR*) connected to its ligand *ergocalciferol* (Vitamin D2). In addition, we found the Estrogen Receptor 1 (*ESR1*) related to the steroid *estradiol* and its derivatives *estradiol acetate* and *estradiol cypionate* (see Table 1 and S1 Table). We also detected a high semantic similarity between steroids that activate the androgen receptor (AR) (e.g. *oxymetholone*, *oxandrolone*, *nandrolone*, *fluoxymesterone*, *oxymetholone*, and *testosterone)* and the AR mouse gene as well as a connection between *testosterone* and the mouse gene coding for the testosterone transforming enzyme aromatase (cytochrome P450 family 19 subfamily A member 1, *CYP19A1*). An indirect relationship in which the drug increases the expression of the gene is exemplified by the high phenotypic similarity between *testosterone* and the follicle-stimulating hormone receptor (*FSHR*) [28].

**Table 1:** Examples of high-scoring gene-drug associations mentioned throughout the text. Information about the associated phenotypic similarity score and the precision (%) based on the benchmarking results with direct drug targets of STITCH is included. (B) indicates that this drug is a biological.

Gene	Drug	ATC_pharma	Similarity score	Precision (%)
AR	*oxandrolone*	anabolic steroids	0.687	66.7
AR	*oxymetholone*	anabolic steroids	0.614	33.3
AR	*nandrolone*	anabolic steroids|other ophthalmologicals	0.457	14.3
AR	*fluoxymesterone*	androgens	0.449	16.7
AR	*testosterone*	androgens|androgens and female sex hormones in combination	0.431	14.6
AR	*methyltestosterone*	androgens|androgens and female sex hormones in combination	0.361	11.1
CASR	*calcium acetate*	Calcium	0.383	10.0
CBFB	*lepirudin recombinant (B)*	-	0.541	14.3
CBFB	*calfactant (B)*	-	0.501	12.1
CBFB	*alteplase (B)*	antithrombotic agents|other ophthalmologicals	0.426	15.3
CYP19A1	*testosterone*	androgens|androgens and female sex hormones in combination	0.699	50.0
CYP19A1	*dutasteride*	drugs used in benign prostatic hypertrophy	0.410	14.7
DRD2	*aripiprazole*	antipsychotics	0.579	25.0
DRD2	*risperidone*	antipsychotics	0.503	12.1
ESR1	*estradiol acetate*	estrogens	0.637	50.0
ESR1	*estradiolum*	estrogens|hormones and related agents	0.479	14.3
ESR1	*estradiol cypionate*	estrogens|hormones and related agents	0.425	15.3
F13A1	*lepirudin recombinant (B)*	-	0.561	20.0
F13A1	*protein c (B)*	antithrombotic agents	0.43	15.5
FGA	*lepirudin recombinant (B)*	-	0.514	10.7
FGA	*alteplase (B)*	antithrombotic agents|other ophthalmologicals	0.382	10.3
FGG	*lepirudin recombinant (B)*	-	0.564	21.4
FSHR	*goserelin*	hormones and related agents	0.433	12.7
FSHR	*lutropin alfa (B)*	gonadotropins and other ovulation stimulants	0.396	12.6
FSHR	*follitropin beta (B)*	gonadotropins and other ovulation stimulants	0.387	10.7
FSHR	*leuprorelin*	hormones and related agents	0.365	11.1
LEP	*paroxetine*	antidepressants	0.4434	14.5
LEP	*aripiprazole*	antipsychotics	0.435	13.2
LEP	*escitalopram*	Antidepressants	0.397	12.0
LEP	*pramipexole*	dopaminergic agents	0.372	10.3
LEPR	*aripiprazole*	antipsychotics	0.561	20.0
LEPR	*paroxetine*	antidepressants	0.522	11.5
LEPR	*escitalopram*	antidepressants	0.419	15.3
LEPR	*pramipexole*	dopaminergic agents	0.412	15.2
LMNA	*mecasermin (B)*	anterior pituitary lobe hormones and analogues	0.407	14.3
PROKR2	*oxandrolone*	anabolic steroids	0.437	13.5
PROKR2	*oxymetholone*	anabolic steroids	0.432	13.8
RUNX1	*lepirudin recombinant (B)*	-	0.540	14.3
RUNX1	*calfactant (B)*	-	0.486	10.8
RUNX1	*alteplase (B)*	antithrombotic agents|other ophthalmologicals	0.436	13.2
SPTA1	*stavudine*	direct acting antivirals	0.595	25.0
TP53	*tamoxifen*	hormone antagonists and related agents	0.604	33.3
VDR	*ergocalciferol*	vitamin a and d, incl. combinations of the two	0.433	13.7

Indirect gene-drug associations might involve different molecular actions ranging from distant (e.g. gene expression) to closer (e.g. an interaction with a gene product physically bound to the drug target) mechanisms. Hence, we decided to investigate whether our method is able to capture close molecular associations between a drug and the gene product. To that aim, we analysed the distance between the known drug target and the gene product in a protein-protein interaction network. We found that phenotypically similar gene-drug pairs tend to share close molecular mechanisms (see Supplemental Information for details).

Taken together, these results indicate that our scoring scheme enables the detection of shared molecular links between drug targets and phenotypically similar gene products. These molecular connections include direct physical binding of the drug to its target as well as indirect effects such as those influencing transcriptional regulation.

### Pharmacogenetic/genomic interactions

Pharmacogenomics studies highlighted the important role of genetic polymorphism in drug efficacy and adverse effects [7]. Whereas some pharmacogenetic associations involve genetic variations in the physical interacting drug targets (e.g. *DRD2* connected to *aripiprazole* and *risperidone*), others appear to be mediated by more complex and indirect functional associations. The observation of gene-drug pairs involved in pharmacogenetic relationships among the top scoring associations prompted us to investigate in a systematic way if gene-drug pairs involved in pharmacogenetic interactions are also enriched in phenotypically similar gene-drug pairs. To test this hypothesis, we utilized the associations annotated in Pharmacogenomics Knowledge Base (PharmGKB) [29]. PharmaGKB contains different types of pharmacogenomics relationships, including the “pheno” and “clinical” associations (see Materials and Methods for details). The “pheno” connections link a gene variant and an affected phenotype, whereas the “clinical” links are manually curated annotations of clinically relevant pharmacogenetic variant—drug pairs. We tested if our scoring scheme can detect gene-drug links resulting from these types of annotations by calculating the enrichment over random (lift) for each relation type. The gene-drug pairs with high phenotypic similarity showed a strong enrichment over random (over 100) for the phenotype annotations and even stronger enrichment of 250 for the clinical associations (Fig 2c).

A known gene-drug pair involved in pharmacogenetic associations among phenotypically similar pairs includes for example the dopamine receptor 2 (*DRD2*) connected to antipsychotics. In the *DRD2* gene, the SNP rs1799978 is a significant predictor for the response to the antipsychotic *risperidone* [30]. Molecularly more distant pairs related to genetic variations on drug response are exemplified by the link between the leptin receptor (*LEPR*) and antipsychotics. In particular, the *LEPR* Q223R polymorphism is significantly associated to obesity in women treated with atypical antipsychotic drugs [31]. Pharmacogenetics is a newly evolving field and consequently, many pharmacogenetic interactions are not known. This might cause an underestimation of the performance of the method to retrieve these interactions. In this context, a literature survey revealed the presence of pharmacogenetic associations among highly similar gene-drug pairs. For example, mutations in the *TP53* gene, which exhibited a high phenotypic similarity to an antagonist of the estrogen receptor *tamoxifen*, have been shown to be a significant predictor of poor response in tamoxifen-treated patients [32]. This example illustrates the potential of the method to reveal novel genes involved in pharmacogenetic interactions.

### Causal drug target-side effect relationships

**Fig 3:**
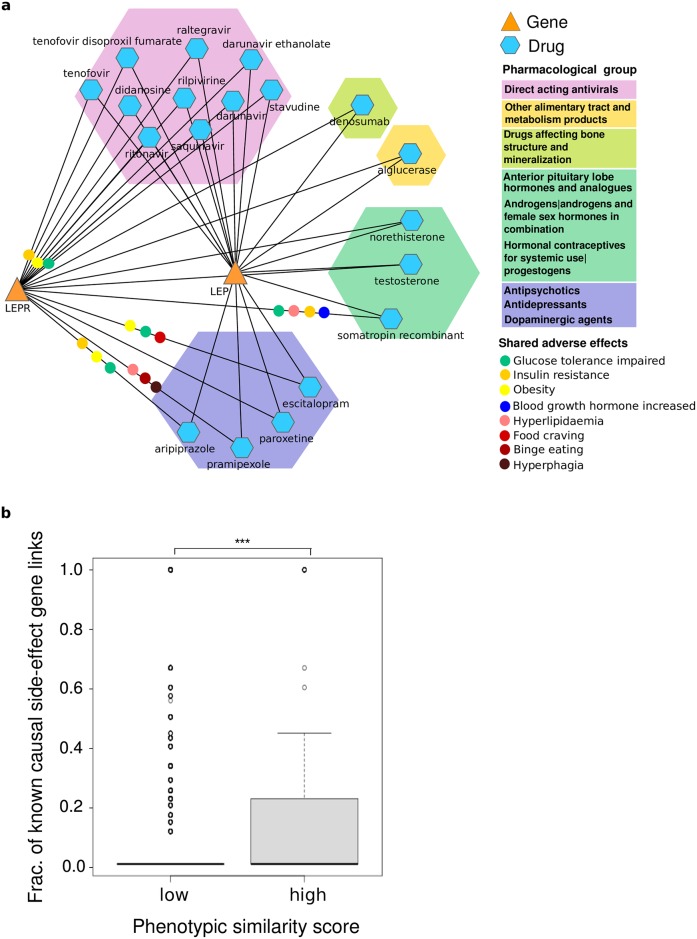
Detection of protein-side effect relationships by the phenotypic similarity approach. a) Network of shared associations between leptin and the leptin receptor and examples of utilized adverse effects (coloured dots). The coloured hexagons indicate the pharmacological subgroup of the ATC classification system. b) Boxplot of the fraction of causal protein-side effect links of low vs high scoring associations utilized in the scoring scheme. The *** denote that this fraction is significantly (P-value = 5.88E-10, Wilkoxon ranksum test) larger in the gene-drug pairs having a high similarity compared to the low scoring ones.

Since drugs and genes sharing similar phenotypes are likely to be molecularly related [24], we reasoned that these shared phenotypes might give valuable insights into protein-side effect relationships and explain the molecular causes of drug adverse effects. For example, the cancer related phenotypes (e.g. “adenocarcinoma”, “malignant soft tissue neoplasm“, and “sarcoma“) linked to *tamoxifen* treatment as well as to mice with impaired *TP53* protein function might be due to the effect of the drug on the function/activity of p53 protein, for example, via a cooperative effect with its target, the estrogen receptor [33]. Similarly, the metabolic effects shared by drugs and mice harbouring defective leptin (*LEP*) or leptin receptor (*LEPR*) could be related to the action of these drugs on the proteins coded by *LEP* and *LEPR* genes. For example, “obesity“, “insulin resistance”, and “glucose tolerance impaired” are phenotypes that atypical antipsychotics as well as antivirals share with *LEP* and *LEPR*. These effects might be caused by the direct or indirect influence of the drugs on these proteins (Fig 3a).

To systematically test if the set of side effects most similar to the gene phenotype reveal causative connections between the gene product and the side effects, we compared these terms to a manually curated dataset of previously reported side effect-protein relationships [18]. In particular, we compared the fraction of known gene-side effect relationships among the shared side effects (see Materials and Methods for details) of high semantic similarity pairs to the corresponding fraction of low scoring pairs. We found that this fraction is significantly (5.88E-10, Wilkoxon ranksum) larger in the gene-drug pairs having a high semantic similarity (Fig 3b).

These findings show that investigating the specific side effects leading to a high overall phenotypic similarity can reveal causative connections between the gene product and the side effects. Thus, our method is capable of providing hypotheses about the molecular mechanisms leading to adverse drug effects.

### Interactions with biologicals

Biologicals are recently gaining the attention of pharmaceutical companies as they open new avenues for targeting non-druggable proteins, that is, proteins that do not bind chemicals naturally. Thus, we tested if our method can also be applied to detect molecular associations of biologicals and shed light onto their side effects. For that, we manually analysed the relationships of the 51 biologicals with mouse genes in our set of top-scoring associations (see S2 Table).

**Fig 4:**
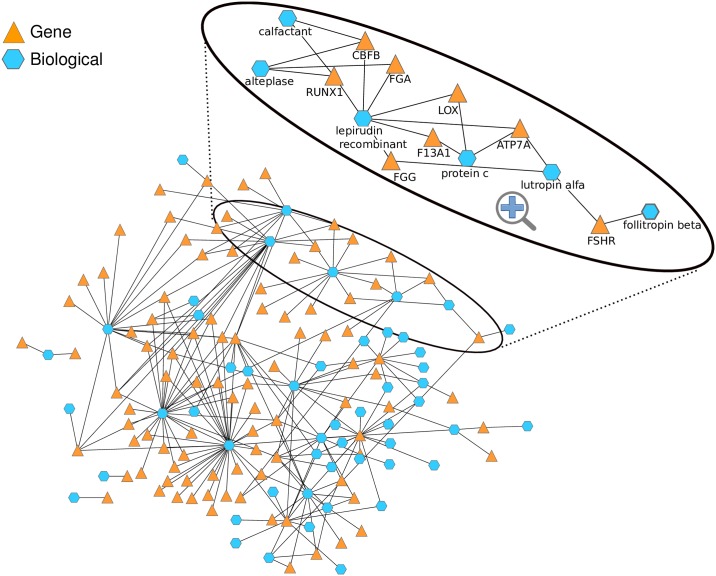
Network of high scoring biological-gene associations. The network of high scoring biological-gene connections is shown and some associations that are discussed in more detail in the manuscript are highlighted in a zoom-in. The follicle stimulating hormone receptor is e.g. connected to *follitropin beta*, a recombinant form of follicle stimulating hormone (FSH) or genes encoding for proteins that are members of the coagulation cascade are linked to anticoagulants like *lutropin alpha*.

Among these associations we found known relationships between biologicals and their protein interaction partners including the link of *follitropin beta*, a recombinant form of follicle stimulating hormone (*FSH*) to its receptor *FSHR* (see zoom-in of Fig 4 and Table 1). We also detected obvious indirect associations between biologicals and the human orthologues of phenotypically similar mouse genes. For example, coagulation related proteins (e.g. alpha and gamma chain of fibrinogen (*FGA*, *FGG*) and coagulation factor XIII (*F13A1*) are connected to *lepirudin recombinant*, a recombinant hirudin derived from yeast cells and a direct thrombin inhibitor (Fig 4 zoom-in and Table 1).

### Experimental validation

All the evidence presented above show that our method can detect drug-target associations as well as gene-drug links resulting from pharmacogenetic studies and provides hypotheses on the molecular mechanisms behind adverse drug effects. To seek for experimental evidence on the phenotypically similar drug-target associations, we first compared our high scoring associations with the hits of *in vitro* assays of ToxCast project [34], which recently systematically tested more than 1,800 compounds in 821 *in vitro* assays. This database contains *in vitro* activity information for 14,238 gene-drug pairs analyzed here, comprising the activity of 141 drugs on different assays related to 263 genes (see Materials and Methods for details). We found experimental information for 38 of the high scoring pairs (S3 Table). These pairs are clearly enriched in associations with experimental support (4-fold, see S8 Fig), with 50% of these pairs appearing as hits of in vitro assays. These results therefore provide experimental support for the phenotypically similar gene-drug associations.

**Fig 5:**
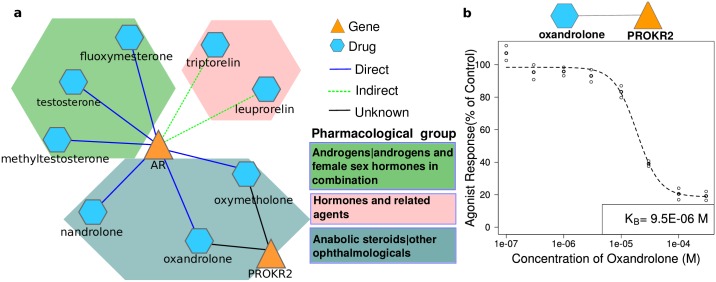
Experimental validation. a) Network of high scoring associations around the PROKR2 gene. PROKR2 is linked to the anabolic steroids *oxandrolone* and *oxymetholone*. The androgen receptor (AR) exhibits a high phenotypic similarity to known direct (blue) and indirect (green) targets of AR. The coloured hexagons indicate the pharmacological subgroup of the ATC classification system. b) Dose response curve of *oxandrolone* on the PKR_2_ antagonistic assay. Two replicate measurements, their average and the fitted dose-response curve are shown.

In order to present also experimental evidence on the newly discovered drug-target associations involving proteins that have not been extensively screened for ligands, we sought for unknown connections of a drug and a mouse gene for which a functional assay for the encoded protein is commercially available. Among these connections, we found the intriguing link between two derivatives of male hormone dihydrotestosterone, *oxandrolone*, and *oxymetholone* and the gene encoding for prokineticin receptor 2 (PROKR2). Prokineticin receptor 2 has recently been implicated for the first time in the binding of small molecules [35–38]. As *oxandrolone* shows a slightly higher phenotypic similarity to *PROKR2* than *oxymetholone*, we decided to investigate if *oxandrolone* has a functional effect on prokineticin receptor 2 protein (PKR_2_). We experimentally tested the possible activity of *oxandrolone* on the receptor in an agonist and an antagonistic functional assays of HEK-293 cells expressing PKR_2_ [39] (see Materials and Methods). We first tested a single concentration of *oxandrolone* (1.0E-05M) on both assays and observed activity on the antagonist assay. We then determined the dissociation constant (K_b_) value (9.5E-06 M) of the *oxandrolone* antagonistic activity in a dose-response curve (Fig 5). To rule out the possibility of a non-specific effect of *oxandrolone* on Ca^2+^ concentrations, *oxandrolone* was tested at high concentrations on HEK-293 cells expressing PKR_2_ in the absence of the known stimulant PK2 (S9 Fig). In these conditions, *oxandrolone* showed no interference with Ca^2+^ mobilization, demonstrating its specificity on PKR_2_ signalling. The experimental validation of the antagonistic effect of *oxandrolone* on PKR_2_ proves that our method is able to detect novel drug-target interactions, further reinforcing the applicability of our method to elucidate drug action.

In summary, we have shown the potential of our novel extended similarity scoring system to unravel molecular mechanisms underlying drug treatment leading to unwanted side effects. Not only known genes encoding for drug targets exhibit a high semantic similarity to their associated drugs, but also protein interaction partners of the known targets do so. Our approach is moreover able to discover gene-drug connections involved in pharmacogenetic interactions, protein-side effects links and associations with biologicals. We furthermore proved experimentally that our method can detect novel drug-target interactions.

## Discussion

In this work, we have analysed the similarity of phenotypes resulting from drug treatment (side effects) and from gene perturbations in mouse models. We showed that our extended semantic similarity approach can elucidate molecular mechanisms that translate drug influence into phenotypic effects giving insights into intended (on-targets) and unintended (off-targets) interactions. The detailed analysis of the phenotypes shared by gene-drug pairs exhibiting a high similarity revealed valuable insights into causative connections between drug targets and side effects. We validated the potential of our approach to detect known drug targets, genes involved in pharmacogenetic associations as well as connections to biologicals and experimentally proved the applicability of our method to detect novel drug-target physical interactions.

Phenotypically similar gene-drug pairs exhibit a strong enrichment in known direct as well as in indirect drug-target relationships (Fig 2b). The proximity of the human orthologue of the mouse gene to the known drug target in the PPI network confirms that our approach detects links between drugs targets and functionally related proteins (S6 Fig). This implies that proteins encoded by genes that share similar phenotypes with a drug are likely to physically interact with that drug’s target or cooperate with it in the same pathway.

The high phenotypic similarity between *LEP/LEPR* and drugs for the nervous system including the dopamine agonist *aripiprazole* and the selective serotonin inhibitors *paroxetine*, *pramipexole* and *escitalopram* further shows the potential of our method to detect non-obvious associations. The shared phenotypes of *aripiprazole* and mice with impaired *LEP/LEPR* function like “obesity”, “insulin resistance” and “glucose tolerance impaired” (Fig 3a) point to alterations of leptinergic signalling by the antipsychotic *aripiprazole*. The pharmacogenetic association of *LEPR* and *LEP* [31] to obesity caused by antipsychotics additionally confirms this. Similarly, the side effects that *LEP* and *LEPR* share with selective serotonin inhibitors such as “food craving”, “binge eating”, “hyperphagia” and “obesity” (Fig 3a) suggest a connection between serotonin signalling and leptin. Indeed, recent studies show that the regulation of appetite by leptin takes place for the most part through inhibition of serotonin synthesis and release by brainstem neurons [40, 41]. These results show that our scoring scheme gives valuable insights into complex mechanisms leading to adverse effects of drugs.

We have demonstrated that our method predicts also molecular associations with biopharmaceutical drugs, where the market has gained a very high and still growing value in recent years [42] and whose adverse effects are often not fully understood. For example, the recombinant form of follicle stimulating hormone (FSH), *follitropin beta*, shows a high phenotypic similarity with mouse models with perturbed *FSHR* gene (Fig 4, Table 1). This association is further confirmed by pharmacogenetic associations in PharmGKB. Consistent with the role of gonadotropin releasing hormone (GnRH) in the release of FSH, we find *FSHR* also associated to *goserelin*, an injectable GnRH superagonist, and to *leuprorelin*, a GnRH analog (Table 1). In line with this association, the stimulation of FSH by GnRH leads to increased levels of testosterone, progesterone, and estradiol. Interestingly, this mechanism is also clearly reflected by the phenotypic traits that *FSHR* shares with *leuprorelin* and *goserelin* that include "Blood testosterone decreased", "Oestradiol decreased", "Progesterone abnormal" and "Blood oestrogen abnormal".

Intriguingly, the steroid *oxandrolone* exhibits a high semantic similarity to its known receptor AR as well as to PROKR2 (Fig 5 and Table 1). We hypothesized that the observed phenotypic similarity between *oxandrolone* and PROKR2 could result from indirect effects e.g. via a functional connection between AR and PROKR2 or, alternatively, from the effects of the drug on the PKR_2_ pathway. To precisely determine the link between *oxandrolone* and PROKR2, we tested the activity of the drug on *in vitro* functional assays and observed an antagonistic effect of *oxandrolone* on PKR_2_ signalling. We showed that this effect is specific of PKR_2_ signalling (see S9 Fig). Furthermore, it is independent from the AR target, since HEK-293 cells do not express AR. These results indicate that the detected antagonistic activity is either mediated via a physical interaction of *oxandrolone* with PKR_2_ or through the interference with downstream proteins of the PKR_2_ pathway. The direct binding of *oxandrolone* to the G-protein coupled receptor (GPCR) protein PKR_2_ is plausible, since several research studies support the involvement of GPCRs on non-genomic effects of androgens and other steroids [43]. These fast effects of steroids are not mediated by the classical transcriptional effects, but through interaction of steroids with their target receptors, which also include GPCRs.

Despite the relative low affinity of *oxandrolone* to PKR_2_, its plasma concentration is compatible with a clinically relevant interaction of *oxandrolone* and PKR_2_. At *oxandrolone* therapeutic dosages (10 mg), its average plasma concentration (417 ng/ml) is in the micromolar range (1.6 μM) [44] and is expected to be even higher in weightlifters and bodybuilders who chronically administer it at supraphysiologic doses [45].

In addition, the physiological effects of a PKR_2_ antagonist as well as those observed in individuals carrying *PROKR2* gene mutations are in concordance with the clinical effects of *oxandrolone* being mediated via PKR_2_ signalling. The clinical effects observed after *oxandrolone* treatment include low levels of luteinizing hormone (LH), GnRH and testosterone [46, 47]. Similarly, a PKR_2_ antagonist (3Cl-MPL) has been shown to blunt circulating luteinizing hormone (LH) levels in mice [48]. Moreover, mutations in *PROKR2* lead to GnRH-deficiency and more specifically to Kallmann syndrome, a disease characterized by hypogonadism, a decreased functional activity of the gonads [49]. Interestingly, mutations in PROKR2 linked to Kallmann syndrome have been shown to impair Ca^2+^ release in HEK293 cells [50, 51], which is consistent with the effect of *oxandrolone* on PKR_2_ in the same cell lines detected herein. However, the effects of *oxandrolone* are not clearly distinguishable from those expected as AR agonist, which also include the influence on LH and GnRH levels [52]. Whether the clinical effects of *oxandrolone* are mediated by the antagonistic activity of *oxandrolone* on PKR_2_, via its activity on the potent target AR or via collaborative effects clearly requires further experimental investigation.

Interestingly, *oxandrolone* causes less virilising effects and also less adverse effects than the natural AR agonist testosterone [53]. Although these effects have been ascribed to the inability of *oxandrolone* to aromatise to estradiol [54], in the light of this new finding, the contribution of PKR_2_ activity of the drug to the decrease of steroid levels deserves further investigation.

Taken together, we could experimentally verify a formerly unknown (direct or indirect) interaction between the GPCR PKR_2_ and *oxandrolone*, which further sheds light into the clinical effects of *oxandrolone* treatment.

The potential of our method was further confirmed experimentally using the results of *in vitro* assays of the ToxCast project. We observed that phenotypically similar gene-drug pairs are strongly enriched in pairs where the drug is active on *in vitro* assays of the protein target encoded by the gene. The results of these assays confirmed associations annotated in the drug target datasets analyzed herein such as *DRD2* and *haloperidol* or *VDR* and *ergocalciferol* and provide experimental support for new ones including *TP53* and *azathioprine* (see S3 Table).

We have demonstrated that our approach is also able to detect genes responsible for the variation on drug response as indicated by the strong enrichment over random of gene-drug associations from PharmGKB (Fig 2c). Recent studies revealed the strong impact of genetic variations on an individual’s response to drugs [55]. However, the majority of the genetic variants responsible for the observed variability on drug response in the population remain to be elucidated. Our method could guide future pharmacogenomic studies by proposing a prioritized list of candidate genes involved in drug response in an analogous manner to gene prioritization used for genome-wide association studies of (rare or multifactorial) diseases [19, 56–58]. This may have important implications in personalized treatment decisions helping to improve drug efficacy and safety.

Although we have shown that our approach can detect many meaningful gene-drug connections based on *in vivo* phenotypic information, it has also limitations inherent to the cross-species comparison. Mutations in genes in mice do not necessarily have the same effect in human and associations involving species-specific (mouse or human) genes or gene families may not be detected by our method. In addition, drugs can act differently in different species, for example due to differences in drug metabolizing enzymes [59]. Terms commonly used as side effect descriptions, such as headache, may not be detected as phenotypic feature in mice. Moreover, the mapping of MPO-annotated phenotypic traits to the MedDRA vocabulary leads to a loss of information among the phenotypes linked to mouse genes. As a consequence, the number of the genes for which we could detect a sufficient phenotypic similarity to drugs is reduced.

Using a stringent mapping procedure, we could translate with high confidence 26% of the mouse phenotypic descriptors utilized as gene annotations in MGI to MedDRA (S1–S4 Files). Interestingly, the coverage of terms mapped to MedDRA terms per mouse gene was higher (29%) than the total number of unique MPO terms mapped to MedDRA. This is explained by the higher likelihood of MPO terms representing frequently observed mouse phenotypic traits to be translated into MedDRA (S1b Fig). This reduces the impact of the loss of phenotypic information in the approach.

We chose to code the phenotypes in the MedDRA terminology over other widely used human clinical ontologies such as HPO to optimally capture information of the effects of drugs in human. MedDRA is intended to be used in the pre- and post-marketing phases of the medicines regulatory process covering also adverse drug reactions [25]. Thus, MedDRA meets our aims better than HPO, which is tailored to cover phenotypic abnormalities of human diseases [60].

Since drug side effect information is recorded from populations with heterogeneous genetic backgrounds, we opted to aggregate phenotypic traits resulting from different genetic perturbations in mice of the same gene (e.g knock-outs, knock-ins or SNPs) and did not differentiate between distinct genetic backgrounds. This has the advantage of enriching the number of phenotypic descriptions linked to genes. In the future, more detailed phenotypic data of genes and drugs would allow the development of more discriminative tools to predict for example agonistic or antagonistic effects of the drug on the gene product and specific genetic variants modulating drug response. This information could include the frequency of occurrence of a side effect per specific drug, additional readouts of the drug action or gene attributes such as metabolic profiles.

Taken together, the results presented here show that our semantic similarity approach is suited to detect mice models mimicking drug phenotypes with high precision and accuracy (see S3c–S3f Fig), allowing determining 1338 phenotypic associations connecting 214 genes and 394 drugs of diverse indication areas. The results of this work demonstrate that the phenotypic similarity between drugs and genes gives valuable insights into molecular mechanisms of drug treatment. The knowledge about relationships between drugs and genes has important implications in personalized treatment decisions, as considering drug mode of action and the genetic predisposition of a patient could circumvent drug inefficacy and adverse effects. Even effects resulting from treatment with combination of drugs may be anticipated if a better understanding of drug mode of action is obtained. Moreover, this information could be used for drug repurposing, because novel drug-target interactions may provide insights for the application of marketed drugs to new indications.

We prove that comparing drug side effects and mouse phenotypic traits reveals insights into drug mode of action. Gene-drug pairs exhibiting a high phenotypic similarity are enriched in known direct and indirect drug-target relationships. Our systems biology approach moreover extends the knowledge about the molecular mechanisms leading to unwanted side effects and about genetic variation influencing drug response. We furthermore provide *in vitro* evidence for the potential of our approach to detect drug-target associations. The experimental validation of a novel drug-target interaction enabled us in addition to get insights into molecular mechanisms of *oxandrolone* treatment.

Thus, this analysis improves drug therapy by advancing the understanding of modes of drug action, adverse effects and genes involved in pharmacogenetic interactions. This may help to find new therapeutic applications for drugs or aid in personalized treatment decisions.

## Materials and Methods

In order to detect relationships between drugs and genes, we calculate the phenotypic similarity of drug side effects and phenotypic traits from gene perturbations in mice.

### Drug phenotypic information (side effects)

We extracted drug phenotypic information from our in-house drug repository which contains 3987 unique side effects associated to 1667 drugs (155,973 pairs) as previously described [26]. The side effect data was parsed from public documents directed at health care professionals or the public such as drug labels, monographs or assessment reports. We annotated the phenotypic information employing the Medical Dictionary for Regulatory Activities (MedDRA), a medical terminology intended to describe e.g. diagnoses, symptoms and signs, adverse drug reactions and therapeutic indications [25]. Terms of this terminology were collected from diverse sources like the World Health Organization's (WHO) adverse reaction terminology, Coding Symbols for a Thesaurus of Adverse Reaction Terms (COSTART) and International Classification of Diseases (ICD) 9 and are maintained, further developed and distributed by the Maintenance Support Services Organisation. MedDRA is organized hierarchically which allows us to compute the semantic relatedness of the terms in this ontology. We used an adapted four-level hierarchy of MedDRA suited for semantic reasoning described previously [26]. In this adapted version of MedDRA, we merged the fifth and the fourth level of MedDRA, because there is no clear hierarchical relationship between these two levels. In addition, as described in [26], we integrated 59 Standardized MedDRA Queries (SMQs), which represent groups of terms across the entire ontology to a defined medical condition. These modifications make MedDRA applicable to measure the semantic similarity of phenotypes.

### Phenotypic information from single gene perturbations in mice

#### Mapping of Mammalian Phenotype Ontology terms to MedDRA

In this section, we describe in detail the procedure we followed to map mouse phenotypes from Mouse Genome Informatics (MGI) [61] encoded in the Mammalian Phenotype Ontology (MPO) [62] to the MedDRA terminology [25] (see also [23]). To obtain the set of gene-phenotype annotations encoded in the MedDRA terminology, we made use of several files given by MGI with information of phenotypes of mice strains. These files are described in the following paragraphs and are provided as supplemental information.

The file MGI_PhenoGenoMP.txt (S1 File, accessed in April 2012) contains information about the allelic composition and the genetic background of the mice strains, MGI marker accession ID and the corresponding phenotypes coded in the MPO. MGI_Coordinate.txt (S2 File, accessed in April 2012) includes the mapping of the MGI marker accession ID to the Ensembl Gene ID. VOC_MammalianPhenotype.txt (S3 File, downloaded from MGI in April 2012) contains all terms of the MPO.

We first mapped MPO terms to the MedDRA terminology. For that, we mapped the terms of the vocabulary of the MPO (from S3 File) to the UMLS Metathesaurus, where MedDRA is integrated, with the help of the MetaMap application (http://mmtx.nlm.nih.gov) [63]. This application from the National Library of Medicine maps biomedical text to the UMLS Metathesaurus using natural language processing. The MetaMap algorithm parses a given text (here the terms of the MPO) into simple noun phrases. Then, for each noun, MetaMap generates variants consisting of the noun itself and its acronyms, abbreviations, synonyms, derivational variants and inflectional and spelling variants derived from the SPECIALIST lexicon and a supplementary database of synonyms [64]. This set of terms is then evaluated against the terms in the UMLS Metathesaurus by calculating the weighted average of four metrics: coverage and cohesiveness, centrality (involvement of the head) and variation (an average of inverse distance scores, which differ for spelling variants, for inflections, for synonyms or acronyms/abbreviation and for derivational variants) [63]. This results in a mapping from MPO to terms in MedDRA that are linked to concepts of the UMLS Metathesaurus. Each mapping is associated to a normalized value between 0 and 1,000, with 0 indicating no match at all and 1,000 indicating an exact match. Those MPO terms with an exact match (score 1000) to MedDRA terms were assigned automatically. Non-exact MPO-MedDRA mappings associated with a high scoring match (score> 865) were manually curated, ensuring a high-quality mapping. Mappings with scores lower than 865 contained a high number of false positives. We, therefore, manually chose this cutoff, in order to assure a high quality of mapped terms (see also S4 File containing the used MPO terms mapped to MedDRA as described below).

Subsequently, we assigned gene names to MGI markers with phenotypic information in the file MGI_PhenoGenoMP.txt utilizing the association to the Mouse Ensembl Gene ID in the MGI_Coordinate.txt file (S2 File). Then we designated the human orthologs of the mouse genes with the Human Ensembl IDs provided by Ensembl BioMart (accessed in Dec. 2011). All MPO phenotypes of the MGI markers linked to the same Human Ensembl Gene ID were assigned to the gene. This allowed mapping 1,939 MPO terms out of 7,051 terms originally assigned to genes in the MGI repository (S1 File) to MedDRA. For 85% (30,811 out of 36,157) of the MPO terms mapped to MedDRA, we could assign a MedDRA term corresponding to an exact match or manually curated mappings (using the webportal MetaMap). For the remaining 15% of the mouse phenotypes annotations linked to genes (5347 out of 36157 MedDRA terms- human genes) the mapping was established via a MPO super-class, again on the basis of an exact match or manually curated term mapped to MedDRA. The full list of gene annotations in MPO mapped to MedDRA can be accessed in S4 File. If the mapping was done using a super-class of the term, this class is also given in the column “super-class MPO” in addition to the annotated MPO term.

#### Analysis of loss of mouse phenotypic information due to the mapping process

In order to evaluate precisely the loss of information after translating MPO terms to MedDRA, we quantified the proportion of MPO phenotypes as well MPO-mouse genes pairs that were mapped to MedDRA. Out of 7,449 MPO terms representing phenotypes of genetically modified mice in MGI (see S1 File), we mapped 1,939 terms to MedDRA used as annotations (including the annotations of more general terms). In total, we mapped 26% of the mice phenotypic annotations. Interestingly, the percentage of mapped MedDRA-mouse gene pairs was higher (29%, 31,025 MedDRA-mouse gene pairs out of 106,639 MPO-mouse gene pairs) than the percentage of unique MPO terms mapped to MedDRA. This might be explained by the higher likelihood of frequently observed mouse phenotypes to be translated to MedDRA.

To test this hypothesis, we binned the MPO coded mice phenotypes by their frequency of occurrence in the MGI data (bin1: 1, bin2:] 1,5], bin3:] 5,10], bin4:] 10,50], bin5:]50,100], bin6: >100). Analogously, we binned the MedDRA coded side effects by their frequency of occurrence in our in-house drug data (bin1: 1, bin2:] 1,5], bin3:] 5,10], bin4:] 10,50], bin5:]50,100], bin6:]100,500], bin7: >500). Some MedDRA terms occur very frequently as side effect description, therefore we added an extra bin containing side effects terms occurring in more than 500 drugs. Then, for each bin, we calculated the fraction of annotations that could be mapped from MPO to MedDRA (S1b and S1c Fig).

#### Comparison of number of phenotypes linked to drugs and mouse genes

Drugs have many more phenotypic annotations compared to single gene perturbations in mice, which is likely caused by the potential of drugs to influence multiple targets. To rule out the possibility that the larger number of drug side effects is due to a loss of information caused by the mapping procedure from MPO to MedDRA, we examined the number of MedDRA–encoded drug side effects in comparison to the number of phenotypes of mouse genes in the original MPO gene annotations as well as mapped to MedDRA. More specifically, we plotted the number of phenotypes of the 6,509 mouse genes with gene annotations in MPO as well as 5,384 human genes with annotations mapped to MedDRA in comparison to the drug annotations (S1a Fig). In order to attribute the polypharmacological property of drugs, we analyzed the average number of side effects per known direct drug target (given in S5 File) for the 1,000 drugs in our data set with direct drug target information. We observed that drugs tend to have many more annotations than mouse genes, even when compared to the original mice phenotypes from MGI encoded in the mammalian phenotype ontology. This indicates that the higher number of drug phenotypes (side effects) is likely to be caused by the tendency of drugs to influence multiple targets.

### Phenotypic similarity method

#### Information content

We assessed the phenotypic similarity of mouse phenotypic traits and drug side effects using a semantic similarity measure based on the information content (IC) as introduced by Resnik [65]. Analogously to a previous approach comparing drugs to diseases [23], the similarity of two terms was calculated by determining the maximal IC of the common ancestors of these terms (Most Informative Common Ancestor (MICA)). We used an IC that is based on the number of children of a term within the ontology and not on the annotation frequency in order to decrease the influence of annotation bias:

$${\text{IC}}_{\text{term}}=\frac{\text{log}\left(\text{child}\left(\text{term}\right)\right)+\text{1}}{\text{log}\left(N\right)}$$

In this equation, term is a given ontology term, child(term) gives the number of all of its children terms, and N denotes the total number of terms in the ontology. Following this formula, a term with less children is considered as more specific.

#### Weighting scheme

Analogously to the previous approach by Vogt et al. [26], frequency and co-occurrence weights were incorporated to downweight frequent and co-occurring terms, as they have been shown to carry less information about drug mechanism [17]. We defined the frequency weight as the negative natural logarithm of the fraction of drugs or genes the term is annotated to. For the co-occurrence weight, we used the negative natural logarithm of the Jaccard index:

$$J\left(A,B\right)=\frac{\left|A\cap B\right|}{\left|A\cup B\right|}$$

Here, A and B represent the sets of drugs and mouse genes the terms under consideration are annotated to.

#### Semantic similarity scores

The similarity score between one side effect i and one mouse phenotypic trait j was then computed as the product of the IC of the MICA and the minimum of the frequency and co-occurrence weights in order to emphasize phenotypic effects specific in both annotation sets:

$$s_{\text{ij}}={\text{IC}}_{\text{MICA}}\cdot \text{min}\left(f_{i}\cdot c_{i},f_{j}\cdot c_{j}\right)$$

where *f*_*i*_ and *c*_*i*_ refer to the frequency and co-occurrence weights of the compared terms.

Subsequently, the final overall phenotypic similarity between a drug and a gene was calculated as follows: For each side effect the best matching mouse trait was determined as identified by the highest similarity score *S*_*ij*_:

$${\text{best}}_{j}=\text{max}\left(s_{i\text{1}},s_{i2},\ldots ,s_{\text{im}}\right)$$

Then, the individual similarity scores from all best matches for a drug were averaged by calculating the arithmetic mean of all best matching scores.

Analogously, for each mouse trait the side effect yielding the highest similarity score among all side effects of the drug was considered as best match:

$${\text{best}}_{i}=\text{max}\left(s_{j\text{1}},s_{j2},\ldots ,s_{\text{jn}}\right)$$

Thus, only if a drug and a gene share specific effects, they will show a high phenotypic similarity.

“Drug polypharmacology”, that is, the tendency of drugs to bind multiple targets, is a well-known property of drugs [21]. Polypharmacology frequently leads to many diverse side effects [23] that contrast with the lower number of phenotypic traits from mouse models resulting from single gene perturbations (see Fig 1a). This difference cannot be explained by the loss of information due to the MPO to MedDRA mapping, as it is also noted when the number of original MPO terms annotated to mouse genes are compared with the drug phenotypic features (S1a Fig). Thus, the large number of side effects of many drugs can be attributed to their polypharmacological property. Hence, only a subset of side effects should be taken into account in the phenotypic similarity calculation of a single gene to a drug. To that aim, we searched for an optimal number of side effects representing effects of a single target for each drug-gene comparison. To determine this number, we evaluated the performance in detecting known drug targets when using only a subset of the highest scoring side effects-gene phenotypes matches (note that the highest scoring side effects-gene phenotype matches might differ between the different genes compared). We tested a broad range of cut-offs differing in the number of top scoring side effects used for the similarity calculation and obtained the best performance by utilizing the 20 best scoring side effects (see S2a Fig). If a drug has fewer than 20 side effects, then the number of all annotated side effects is taken into account. This threshold also exhibited an optimal performance when retrieving associations from an independent and mutually exclusive dataset of indirect drug-target interactions (S2b Fig). This shows, that the best performance for a cut-off of 20 best scoring side effects for each drug-gene pair is not a result of data over-fitting.

We calculated the arithmetic mean of these 20 top scoring phenotypic features of a drug and all best scoring phenotypic features of a gene for each drug-gene pair. In this manner, our new scoring scheme enabled us to find gene-drug relationships resulting from one drug target while disregarding the side effects originating from other targets.

A gene with very few annotations can score high with a drug if one of the annotations is similar to a side effect by chance. This is more likely to happen when comparing genes with few annotations to drugs with many side effects, resulting frequently in false positives in high scoring pairs. To alleviate this issue, we downweighted associations from genes with low phenotypic information by multiplying the score with the natural logarithm of the number of MedDRA terms annotated to mouse genes. Additionally, we binned the drugs into three bins depending on the number of side effects and weighted them accordingly (low (0–33% quantile): weight 1), medium (33–66% quantile): weight 0.66, high (66%-100% quantile): weight 0.33). Subsequently we normalized the resulting score by the maximum occurring value in order to get a value between 0 and 1.

### Performance measurement

To evaluate the performance of the presented phenotypic similarity scoring scheme we used Receiver Operating Characteristic (ROC), precision, lift and accuracy plots. For all performance plots, the R package "ROCR" [66] was utilized. In the following paragraph we explain the different performance measurements.

Let Y be a random variable representing the known information about the relatedness of a drug-gene pair and *Ŷ* a random variable representing the classification according to the scoring scheme for a randomly drawn sample. φ denotes the positive class and $\bar{\varphi }$ denotes the negative class, respectively. Further, P describes the number of (empirical) positives, N the number of negatives, TP the number of true positives and FP the number of false positives.

#### ROC plots

A ROC plot is created by plotting the true positive rate against the false positive rate at each score threshold. The true positive rate, also known as recall, measures the proportion of actual positives, which are correctly identified as such, it is, thus, a measure for the specificity of a classification system:

$$\text{True positive rate: }P\left(\hat{Y}=\varphi |Y=\varphi \right)=\frac{TP}{P}$$

The false positive rate measures the proportion of false positives among all negative samples:

$$False\;positive\;rate\text{: }P\left(\hat{Y}=\varphi |Y=\bar{\varphi }\right)=\frac{FP}{N}$$

#### Accuracy

The accuracy measures the proportion of true positives and true negatives among the total number of samples:

$$\text{Accuracy: }P\left(\hat{Y}\text{=Y}\right)=\frac{\text{TP}+\text{TN}}{P+N}$$

#### Precision

The precision value is defined as the fraction of true positives among all positives at a certain score threshold:

$$\text{Precision: }P\left(Y=\varphi |\hat{Y}=\text{φ}\right)=\frac{\text{TP}}{TP+FP}$$

The precision, thus, measures the percent of correct positive predictions at a certain score.

#### Lift

The lift value additionally incorporates a random choice model, by considering the precision as well as the probability of obtaining a true value by chance:

$$\text{Lift: }\frac{P\left(\hat{Y}=\varphi |Y=\varphi \right)}{P\left(\hat{Y}=\varphi \right)}=\frac{\frac{P\left(Y=\varphi |\hat{Y}=\varphi \right)P\left(\hat{Y}=\varphi \right)}{P\left(Y=\varphi \right)}}{P\left(\hat{Y}=\varphi \right)}=\frac{P\left(Y=\varphi |\hat{Y}=\varphi \right)}{P\left(Y=\varphi \right)}=\frac{\text{Precision}}{P\left(Y=\varphi \right)}=\frac{\text{Precision}}{P\left(\text{true}\right)}$$

The area under the ROC curve (AUC, given in the manuscript as number truncated after three digits) is a valuable measurement of the overall performance of a scoring scheme independent from the threshold of the score. However, for the classification of millions of drug–gene pairs only the highest scoring ones (those located at the lower left corner of a ROC plot) are relevant. Thus, precision and lift plots, depicting the performance values at each score, are more suitable to show the performance at the high scoring regions.

The function of the lift measurement ($\frac{\text{Precision}}{P\left(\text{true}\right)}$) has the same shape as the precision function, because the probability of randomly obtaining a true value does not depend on the evaluated score. Yet, the lift value has the advantage to also show the enhancement of the predictive power of a scoring scheme in relation to the case where no scoring scheme is applied. For instance, if a benchmark set contains many true values, the precision would be high even without an adequate scoring scheme. The lift value, in contrast, would be low if no suitable scoring was applied. Taken together, the lift value measures the performance of a scoring scheme compared to random expectation.

### Drug-target interactions

We benchmarked our results with known human drug-targets interactions from the STITCH database [27]. We only used associations from curated databases, excluding e.g. relationships based only on textmining and applied a high confidence cutoff of 0.7 for the drug-target relationships. We mapped the drugs by name, including synonyms, to our in house drug dictionary. Moreover, we distinguished direct physical interactions from indirect ones as described in [26]. We created a benchmark set of direct interactions and another one of indirect associations by selecting those genes/drugs where at least one physical or indirect interaction has been reported, respectively. In these benchmark sets we constructed the positive set with all known associations involving those genes and drugs and the negative set by all possible combinations of these drugs and genes. This resulted in 863,074 and 4,118,052 drug gene pairs, respectively. We divided our set of drug-gene associations into high and low scoring ones where the precision in direct associations exceeded 10%, which was true at a score higher or equal to 0.354. This lead to 1338 high scoring associations linking 214 genes to 394 drugs.

### Comparison with existing method

We compared the developed scoring scheme to the one proposed by Hoehndorf et al. [24] by evaluating the performance of the algorithm of Hoehndorf and collaborators using our data.

The semantic similarity scoring scheme proposed by Hoehndorf and collaborators provides two asymmetric scores corresponding to drug-gene and gene-drug pairs. In order to compare our approach with the previous published one [24] using the same annotation framework, we applied the algorithm (https://code.google.com/p/phenomeblast/source/browse/trunk/phenotypenetwork/SimGIC-twosides.cc) proposed by Hoehndorf to our MedDRA framework and phenotypic annotations (see S4 Fig). To calculate the Hoehndorf non-symmetric scores, we utilized our phenotypes of drugs and genes encoded in MedDRA and the information content of the most informative common ancestor within MedDRA as input of the given code. We did not change the code of the provided algorithm, implying a pre-set threshold of at least 7 phenotypes defined in the code (define MINPHENOTYPES 7). Subsequently, we compared the resulting non-symmetric similarity scores (drug-gene and gene-drug pairs) to the scores of our scoring scheme, where we also applied the threshold of 7 phenotypes. We compared the performance of the two approaches using lift and ROC plots (S4 Fig) and our benchmark set of direct drug-target interactions (see section drug-target interactions above). Both performance measurements showed that our approach outperforms the method proposed by Hoehndorf and collaborators.

To calculate the significance of the increase of the ROC plots over the method proposed by Hoehndorf et al., we used the function roc.test from the R package pROC to compare the differences in ROCAUC. We tested the hypothesis that our ROC plot has significantly higher AUC values than each of the assymetric scores proposed by Hoehndorf et al. We observed significantly higher ROCAUC values for our method.

Also the lift plot illustrates that the approach presented here performs remarkably better classifying true positives at high scoring regions.

### Influence of benchmark sets on ROC plot performance

We noticed that the application of Hoenhdorf algorithm to our benchmark set of physically interacting human drug-targets results in lower AUC values than those published by Hoehndorf, who use different benchmark sets [24]. These differences suggest that the benchmark sets have an influence on the performance as measured by the ROC plots. In order to confirm this hypothesis, we evaluated the performance of the scores of our MedDRA-based semantic similarity measurement on two benchmark sets provided by Hoehndorf (in http://phenomebrowser.net/drugeffect-data.tar.bz2). In particular, we benchmarked our approach with the DrugBank (drugeffect-data/positive/drugbank-targets.txt) and STITCH (drugeffect-data/positive/stitch-human-targets-0.7.txt) datasets, which contain associations between gene MGI IDs and drug STITCH IDs reported in the DrugBank [67] and STITCH databases [27], respectively. We first mapped the drugs and genes for which we have phenotypic information to MGI and STITCH identifiers. To map the genes in our dataset to the MGI IDs in these benchmark sets, we utilized the file mousephenotypes-names.txt provided by Hoehndorf et al. containing the MGI marker ID and gene name (in the folder drugeffect-data/input-phenotypes/). We mapped the drug names to STITCH IDs (STITCHORIG:) using a file downloaded from the STITCH website (chemical.aliases.v3.1.tsv, accessed in Dec. 2013). This resulted in 7,262,444 MGI ID-STITCH ID pairs associated with scores from our algorithm. Following the descriptions of Hoehndorf and collaborators, we constructed ROC plots utilizing as positive sets the DrugBank and STITCH benchmark files and all the remaining possible associations of drugs and genes as negative set. The negative set, thus, includes drugs with side effects linked to genes with phenotypic information never occurring in the evaluation dataset of DrugBank or STITCH. This results in a bigger dataset with a higher proportion of true negatives in low similarity regions leading to a better performance in the ROC curve (see S5 Fig), although the interesting top-scoring cases only contribute very little to the overall AUC. These results demonstrate the influence of the different benchmark sets on the ROC plot evaluation performance.

### Interactions with biologicals

Biologicals or biopharmaceuticals are “recombinant therapeutic proteins and nucleic acid based products and in the broader sense also engineered cell or tissue-based products”[68, 69]. In order to identify biologicals within highly similar drug-gene relations, we manually curated the list of drugs that are part of these relationships. In total, we identified 51 biologicals associated with a high phenotypic similarity to 95 genes via 226 connections by our phenotypic similarity measurement.

### Shortest path in Protein-Protein Interaction (PPI) network to known drug target

We extracted protein-protein interactions from the String database [70]. In order to guarantee high confident interactions we selected protein-protein associations using a cutoff of 0.7 [71]. Using this network, we calculated the shortest path for each human orthologue of the mouse gene product in our data set to all drug targets from drugs in our data set. We used the target information provided by STITCH where a direct interaction is reported and annotated for each drug-gene pair the closest distance between a known target of the drug and the human gene product.

### Pharmacogenetic/genomic interactions

To test if our method is able to detect genes involved in pharmacogenetic associations, we compared our results to the data collected in the Pharmacogenomics Knowledge Base (PharmGKB) [29]. PharmGKB includes connections of genes to single drugs, groups of drugs as well as therapeutic classes of drugs. If multiple drugs were annotated simultaneously to one gene, we split these associations and treated each drug-gene pair individually. In order to analyse therapeutic classes of drugs, we classified the drugs in our dataset using the class “pharmacological subgroup” from the Anatomical Therapeutic Chemical (ATC) classification system. Subsequently, we mapped the resulting set via the drug name (including synonyms), if possible, or via pharmacological subgroup otherwise to the files “clinical_ann” and “pheno_ann” of PharmGKB. The “clinical” links are manually curated annotations of clinically relevant pharmacogenetic variant—drug pairs and “pheno” connections link a gene variant and an affected phenotype. The positive set of our investigation consisted of all drug-gene associations from PharmGKB and the negative set of all possible drug-gene combinations of this mapping where no pharmacogenetic interaction was reported in PharmGKB. Altogether we analysed 616.955 and 4.401.175 drug-gene pairs by calculating the enrichment over random (lift) of clinical and phenotypic pharmacogenetic associations in these pairs, respectively. We moreover checked the distribution of the quantity of data points by binning according to the phenotypic similarity score and calculating the natural logarithm of the number of drug-gene pairs per bin (S7 Fig).

### Causal gene-side effect relationships

We checked if the side effects most similar to gene phenotypic traits of the most phenotypic similar drug-gene pairs are enriched in a manually curated dataset of known side effect-protein relationships published recently [19]. For every drug-gene pair, we calculated the fraction of known gene-adverse effect associations among the side effects sharing similar phenotypic traits to the mouse gene under consideration (maximal 20 side effects). Subsequently, we compared the resulting fraction of causal gene-side effect relationships of the high-scoring pairs to the low-scoring ones.

### ToxCast *in vitro* assays

To provide experimental evidence for interactions of phenotypically similar drug-gene pairs, we compared our results to the hits of the ToxCast project [34, 72]. We collected the hit annotations from the file AllResults_hitc_Matrix_141121.csv, where 1860 compounds are tested in 822 assays. Subsequently, we annotated the assays to their intended target and mapped the targets to our set of genes and the compounds to our drug data set. Then, we calculated the precision of the resulting 14,238 drug-gene pairs in relation to their phenotypic similarity score. Furthermore, we manually investigated the 38 high scoring drug-gene pairs, which included 19 experimentally validated associations.

### Experimental validation of a novel drug-target interaction

In order to test the hypothesis that *oxandrolone* interacts with prokineticin receptor 2 (PKR_2_), the antagonistic and agonistic effects of *oxandrolone* were investigated in functional assays of PKR_2_ activity [39]. All the experiments were performed by the company CEREP. *Oxandrolone* was purchased from Sigma-Aldrich and Ehrenstorfer GmbH. In these assays the activity of PKR_2_ was traced measuring the intracellular Ca^2+^ by fluorimetry in HEK-293 cells expressing PKR_2_. In the agonist experiments, Ca^2+^ mobilization after *oxandrolone* stimulation was measured and then the agonist effect of *oxandrolone* was calculated as a % of control response to the known reference agonist PK2 (used at 10 nM concentration). The activity of *oxandrolone* on the antagonist assay was tested after the stimulation of PKR_2_ with the control reference agonist PK2 at a concentration of 2 nM. The antagonist effect was then calculated as a % inhibition of control reference agonist response.

*Oxandrolone* was initially tested in both assays at a concentration of 1.0E-05M. At this concentration, we only detected activity on the antagonist assay (32.3% inhibition of control agonist response). We subsequently quantified the antagonistic effect of *oxandrolone* in a dose-response curve and determined the K_b_ (dissociation constant) value. We fitted the dose-response curve using the R function drm from the package drc (cran.r-project.org/web/packages/drc/index.html) with the four-parameter log-logistic function LL.4.

To rule out the possibility of a non-specific effect of *oxandrolone* on Ca^2+^ concentrations on the antagonist assays, we additionally measured Ca^2+^ using fluorimetry in PKR_2_ expressing HEK-293 cells employing high concentrations of *oxandrolone* (1.0E-05, 3.0E-05, 1.0E-04, 3.0E-04) in the absence of the known stimulant PK2 (S9 Fig).

## Supporting Information

S1 Table Table of high scoring drug-gene associations. We extracted 1338 high scoring associations connecting 214 genes and 394 drugs by cutting at the phenotypic similarity score where the precision in the direct associations exceeds 10%.
(TXT)

S2 Table Table of high scoring drug-gene associations with biologicals. (TXT)

S3 Table Table of high scoring associations with *in vitro* activity information in ToxCast. (TXT)

S1 File MGI_PhenoGenoMP.txt (accessed in April 2012) contains information about the allelic composition and the genetic background of the mice strains, MGI marker accession ID and the corresponding phenotypes coded in the Mammalian Phenotype Ontology. (TXT)

S2 File MGI_Coordinate.txt (accessed in April 2012) contains the association of the MGI marker accession ID to the Ensembl Gene ID and additional gene and sequence related information. (TXT)

S3 File VOC_MammalianPhenotype.txt (downloaded from MGI in April 2012) comprehends the vocabulary of the Mammalian Phenotype Ontology in a tab-delimited format. (TXT)

S4 File Mouse_Human_Gene_MPO_MedDRA.txt contains the phenotypic annotations of mouse genes mapped from the Mammalian Phenotype Ontology to MedDRA. Those MPO terms linked to genes with a corresponding specific MedDRA term are annotated. The MPO terms without a match to a specific MedDRA term were translated, when possible, utilizing the mapped super-classes of these MPO terms. The mouse gene, the human orthologue and the annotated MPO term is given. In addition, we provide the MPO term of the super-class if the mapping was done utilizing the super-class of a term (column 'mapped super-class MPO'). Moreover, the most_specific_MedDRA_code to which the mapping was conducted and its level within the MedDRA hierarchy is presented (HG = 'High Level Group Term', HL = 'High Level Term', LLT/P = ‘Lowest Level Term/Preferred Term’ where LLT/PT represent the most specific level, followed by HL and then by HG).
(TXT)

S5 File S5_DirectTargets.txt contains the benchmark set of physically interacting drug-gene pairs. The CID (the ID from the STITCH database), the drug name and the human Ensembl gene ID and the name of the interacting gene is given.
(TXT)

S1 Fig Evaluation of the mapping from the Mammalian Phenotype Ontology (MPO) to the MedDRA terminology. a) To rule out the possibility that the larger number of drug side effects compared to the number of phenotypes of single gene perturbations in mice is due to a loss of information caused by the mapping procedure, we compared the number of MedDRA–encoded drug side effects (green line) to the number of phenotypes of mouse genes in the original MPO gene annotations (dark blue) as well as mapped to MedDRA (light blue). More specifically, we plotted the number of MPO original annotations of 6509 mouse genes (Genes MP) as well as the number of MedDRA annotations for 5384 mouse genes (Genes MedDRA) sorted by number of phenotypes (x-axis). As drugs influence multiple gene products, we plotted the average number of annotations (y-axis) per direct drug target in our dataset sorted by increasing number of side effects (x-axis). Drugs tend to have many more annotations than mouse genes, even when compared with the original mice phenotypes from MGI encoded in the mammalian phenotype ontology. b) MPO coded mice phenotypes binned by their frequency of occurrence as gene annotations in the MGI data (x-axis). For each individual bin, we calculated the fraction of MedDRA terms that could be mapped from MPO terms (y-axis). This fraction increases with increasing gene annotation frequency of the MPO terms.
(TIF)

S2 Fig Cut-off evaluation for the adaption of the phenotypic similarity approach to account for the polypharmacology property of drugs. We searched for an optimal number of side effects representing effects of a single drug target. To that aim, we tested a broad range of different numbers of side effects-mouse gene phenotype best matches (depicted in different colors) used for the similarity calculation in a benchmark dataset of direct drug-target interactions (a). We obtained the best performance by considering the 20 best scoring side effect-phenotype pairs (yellow line). We confirm the suitability of the cut-off of 20 side effects-mouse phenotype best matches for the drug-gene semantic similarity score calculation by evaluating the different cut-offs using an independent and mutually exclusive benchmark datasets of indirect (b) drug-target interactions.
(TIF)

S3 Fig Precision and Receiver Operating Characteristic (ROC) and accuracy plots for the different cut-offs (in different colors) evaluated for the adaption of the phenotypic similarity approach to account for the polypharmacology property of drugs. The performance in detecting direct (a, c and e) and indirect (b,d and f) drug-target interactions is shown.
(TIF)

S4 Fig Comparison to the semantic similarity method proposed by Hoehndorf [24] in the potential to detect direct drug-target associations from the STITCH database. Hoehndorf et al. calculated two asymmetric similarity scores, a drug-gene (black line) score and a gene-drug score (grey line). Lift plots (left) and ROC (right) are shown comparing the non-symmetric measure of semantic similarity proposed by Hoehndorf and collaborators to the one developed herein (Prinz et al., blue line). We applied this algorithm to drugs and genes part of our benchmark set of direct drug-target interactions. We utilized our phenotypes of drugs and genes encoded in MedDRA and the information content of the most informative common ancestor within MedDRA as input of the algorithm provided by Hoehndorf et al. The code of this algorithm (https://code.google.com/p/phenomeblast/source/browse/trunk/phenotypenetwork/SimGIC-twosides.cc) defines a pre-set threshold of at least 7 phenotypes, which we consequently also applied to our data. The approach developed herein noticeable outperforms the scoring scheme developed by Hoehndorf et al. The non-homogeneous shape of the lift plots in the high scoring regions is due to the decreasing number of data points. Our method results in significantly higher ROCAUC values than the method proposed by Hoehndorf et al to calculate gene-drug and drug-gene semantic similarity scores (P-value = 7.7E-34 and 4.6E-21 respectively).
(TIF)

S5 Fig ROC plots evaluating our semantic similarity method with the benchmark sets provided by Hoehndorf [24] (data downloaded from http://phenomebrowser.net/drugeffect-data.tar.bz2). The performance of the method on the benchmark sets that Hoehndorf and collaborators gathered from STITCH (human) (drugeffect-data/positive/stitch-human-targets-0.7.txt) is shown in light blue and from DrugBank (drugeffect-data/positive/drugbank-targets.txt) is depicted in darker blue.
(TIF)

S6 Fig Distance in the PPI-network of STRING from gene products part of low (high) scoring drug-gene associations to known drug targets. Phenotypically similar drug-gene pairs tend to share close molecular mechanisms, as drug-gene pairs involving a drug target that interacts directly (shortest path distance 0) or through a common protein (shortest path distances 1 or 2) with the protein coded by the human orthologue of the mouse gene are significantly enriched in high semantically similar pairs (P-value = 3.41E-62, Wilcoxon test).
(TIF)

S7 Fig Data distribution of PharmGKB benchmark set in relation to the similarity score. We binned the drug-gene pairs according to their phenotypic similarity and calculated the natural logarithm of the number of drug-gene pairs in the benchmark set of PharmGKB per bin.
(TIF)

S8 Fig Enrichment over random (a) and precision (b) of hits of *in vitro* assays from the ToxCast project. The intended targets of the ToxCast assays were mapped to our set of genes and the compounds to our drug data set. The precision and lift of the resulting 14,238 drug-gene pairs showed the strong tendency of phenotypically similar drug-gene pairs to be a hit in assays of the ToxCast project.
(TIF)

S9 Fig Specificity of the antagonistic activity of *oxandrolone* on PKR_2_. We tested *oxandrolone* in HEK-293 cells expressing PKR_2_ in the absence of PK2, the known ligand of the receptor. We could not detect activity of *oxandrolone* in this assay, even at the highest concentrations tested. This result demonstrates the specificity of the action of *oxandrolone* on PKR_2_ signaling, thereby excluding the possibility of interference through a completely different route.
(TIF)

S1 Text Investigation if the targets of a drug that is phenotypically similar to a mouse gene are close to the proteins encoded by this mouse gene in a protein-protein interaction (PPI) network. (DOC)
