# Supplementary figures and images for: A Novel Drug-Mouse Phenotypic Similarity Method Detects Molecular Determinants of Drug Effects
Source: PLoS Comput Biol. 2016 Sep 27;12(9):e1005111. doi: 10.1371/journal.pcbi.1005111 (PMC5038975; doi:10.1371/journal.pcbi.1005111)

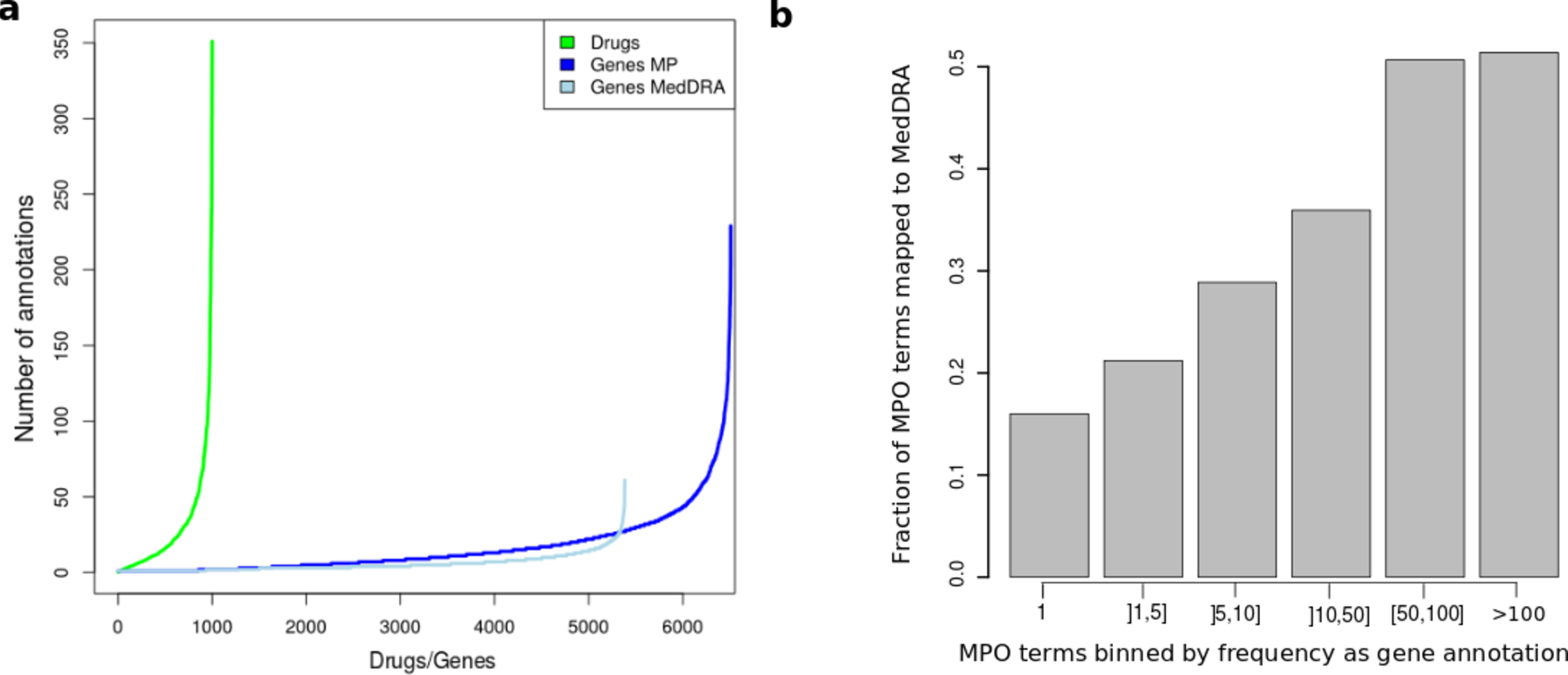

Supplement: S1 Fig — a) To rule out the possibility that the larger number of drug side effects compared to the number of phenotypes of single gene perturbations in mice is due to a loss of information caused by the mapping procedure, we compared the number of MedDRA–encoded drug side effects (green line) to the number of phenotypes of mouse genes in the original MPO gene annotations (dark blue) as well as mapped to MedDRA (light blue). More specifically, we plotted the number of MPO original annotations of 6509 mouse genes (Genes MP) as well as the number of MedDRA annotations for 5384 mouse genes (Genes MedDRA) sorted by number of phenotypes (x-axis). As drugs influence multiple gene products, we plotted the average number of annotations (y-axis) per direct drug target in our dataset sorted by increasing number of side effects (x-axis). Drugs tend to have many more annotations than mouse genes, even when compared with the original mice phenotypes from MGI encoded in the mammalian phenotype ontology. b) MPO coded mice phenotypes binned by their frequency of occurrence as gene annotations in the MGI data (x-axis). For each individual bin, we calculated the fraction of MedDRA terms that could be mapped from MPO terms (y-axis). This fraction increases with increasing gene annotation frequency of the MPO terms. (TIF) [file pcbi.1005111.s009.tif]

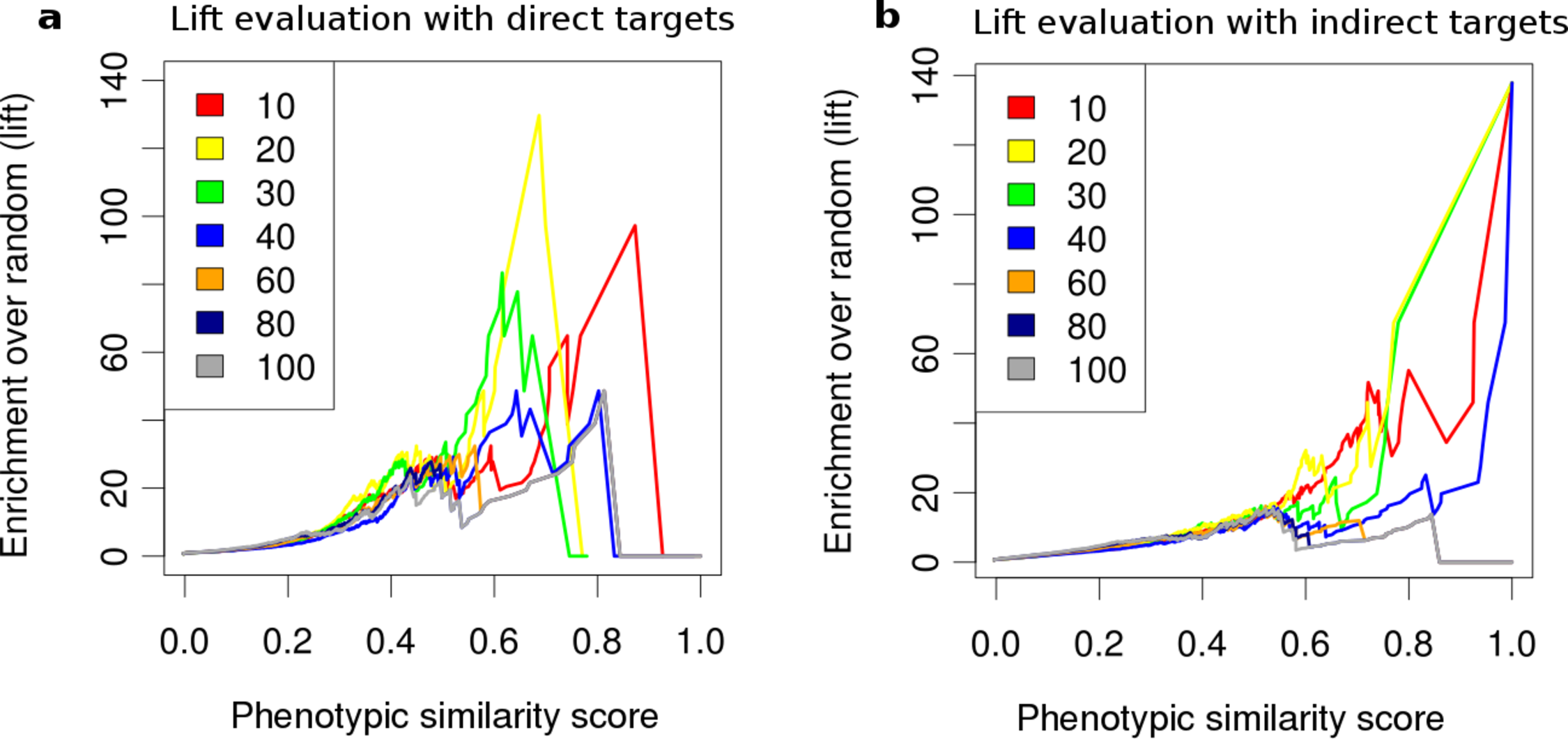

Supplement: S2 Fig — We searched for an optimal number of side effects representing effects of a single drug target. To that aim, we tested a broad range of different numbers of side effects-mouse gene phenotype best matches (depicted in different colors) used for the similarity calculation in a benchmark dataset of direct drug-target interactions (a). We obtained the best performance by considering the 20 best scoring side effect-phenotype pairs (yellow line). We confirm the suitability of the cut-off of 20 side effects-mouse phenotype best matches for the drug-gene semantic similarity score calculation by evaluating the different cut-offs using an independent and mutually exclusive benchmark datasets of indirect (b) drug-target interactions. (TIF) [file pcbi.1005111.s010.tif]

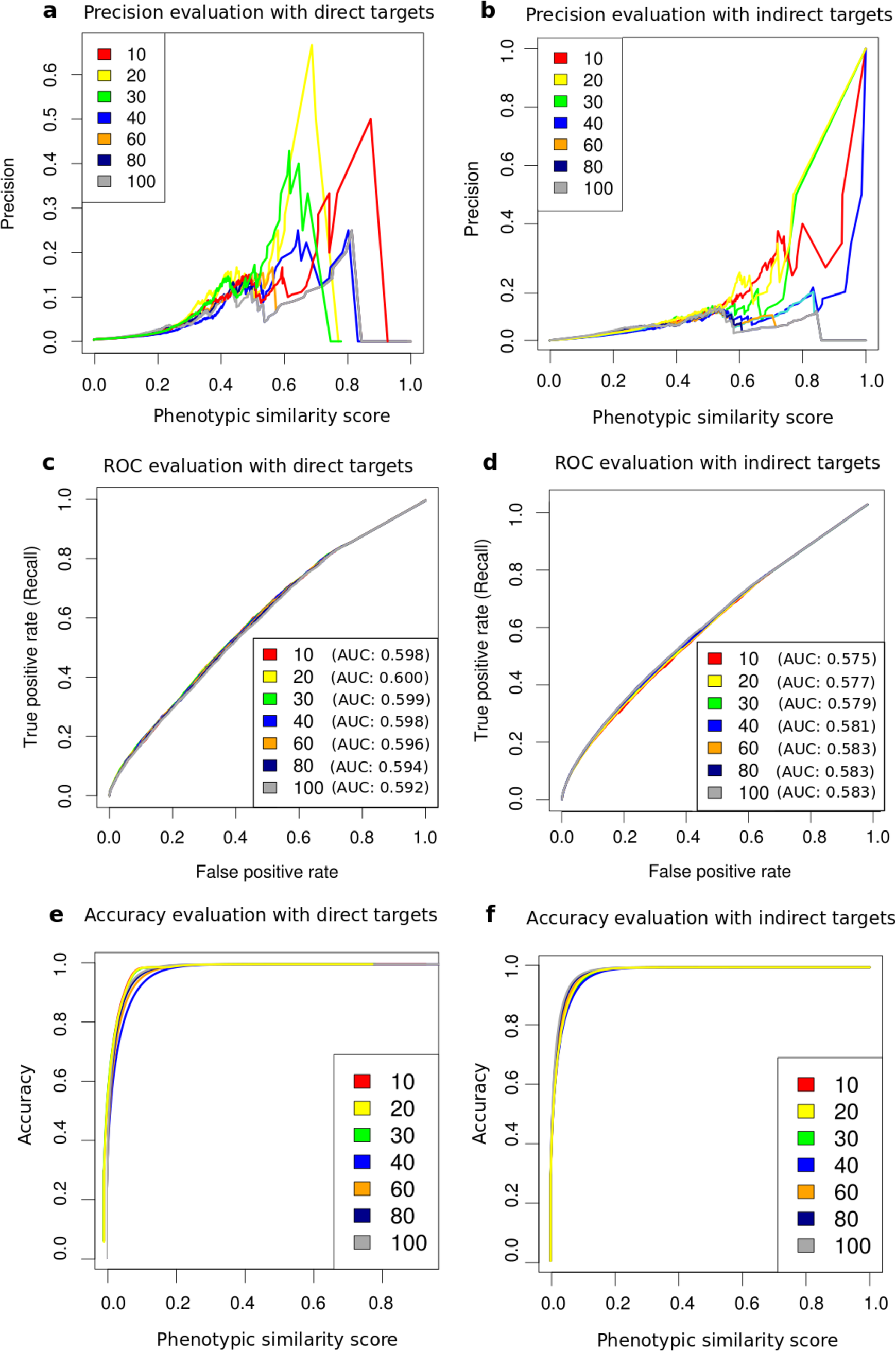

Supplement: S3 Fig — The performance in detecting direct (a, c and e) and indirect (b,d and f) drug-target interactions is shown. (TIF) [file pcbi.1005111.s011.tif]

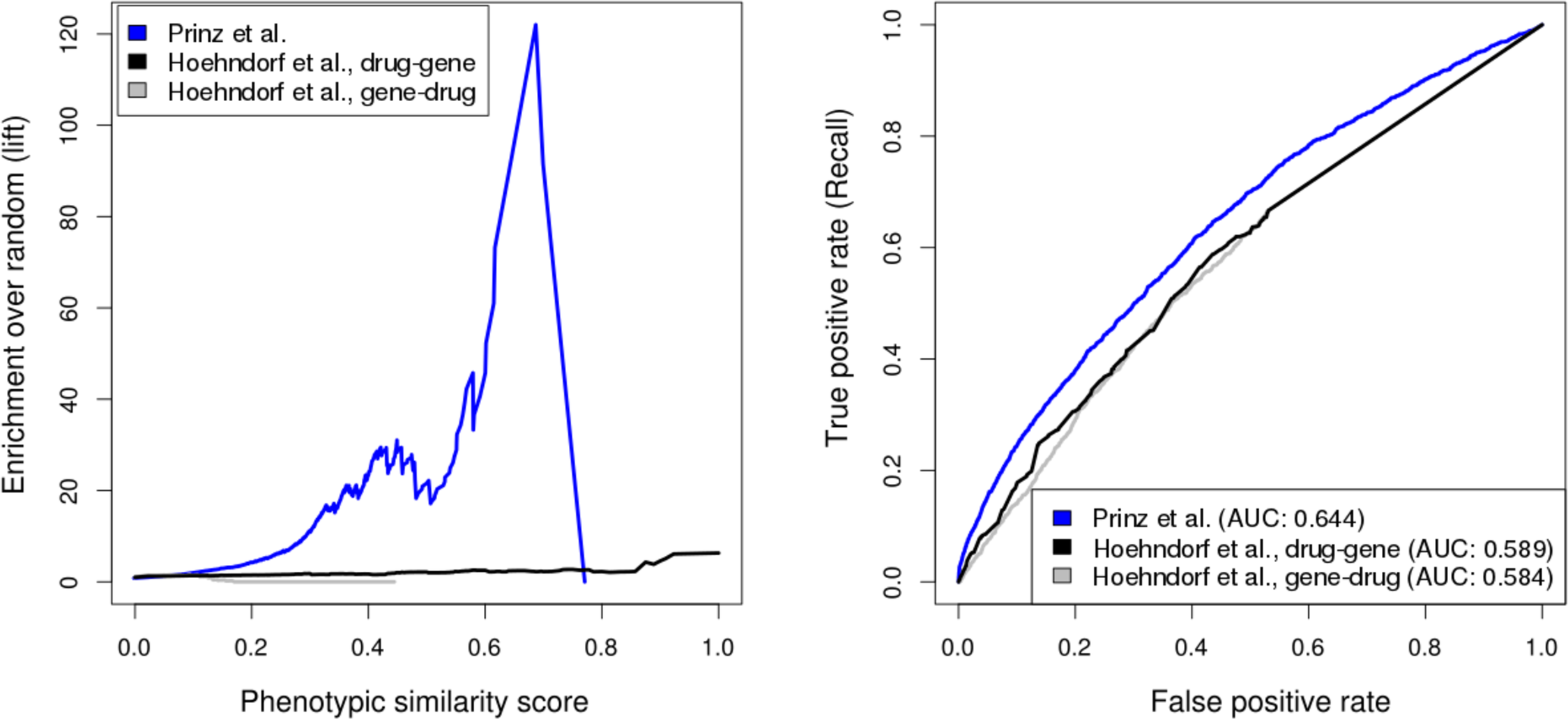

Supplement: S4 Fig — Hoehndorf et al. calculated two asymmetric similarity scores, a drug-gene (black line) score and a gene-drug score (grey line). Lift plots (left) and ROC (right) are shown comparing the non-symmetric measure of semantic similarity proposed by Hoehndorf and collaborators to the one developed herein (Prinz et al., blue line). We applied this algorithm to drugs and genes part of our benchmark set of direct drug-target interactions. We utilized our phenotypes of drugs and genes encoded in MedDRA and the information content of the most informative common ancestor within MedDRA as input of the algorithm provided by Hoehndorf et al. The code of this algorithm (https://code.google.com/p/phenomeblast/source/browse/trunk/phenotypenetwork/SimGIC-twosides.cc) defines a pre-set threshold of at least 7 phenotypes, which we consequently also applied to our data. The approach developed herein noticeable outperforms the scoring scheme developed by Hoehndorf et al. The non-homogeneous shape of the lift plots in the high scoring regions is due to the decreasing number of data points. Our method results in significantly higher ROCAUC values than the method proposed by Hoehndorf et al to calculate gene-drug and drug-gene semantic similarity scores (P-value = 7.7E-34 and 4.6E-21 respectively). (TIF) [file pcbi.1005111.s012.tif]

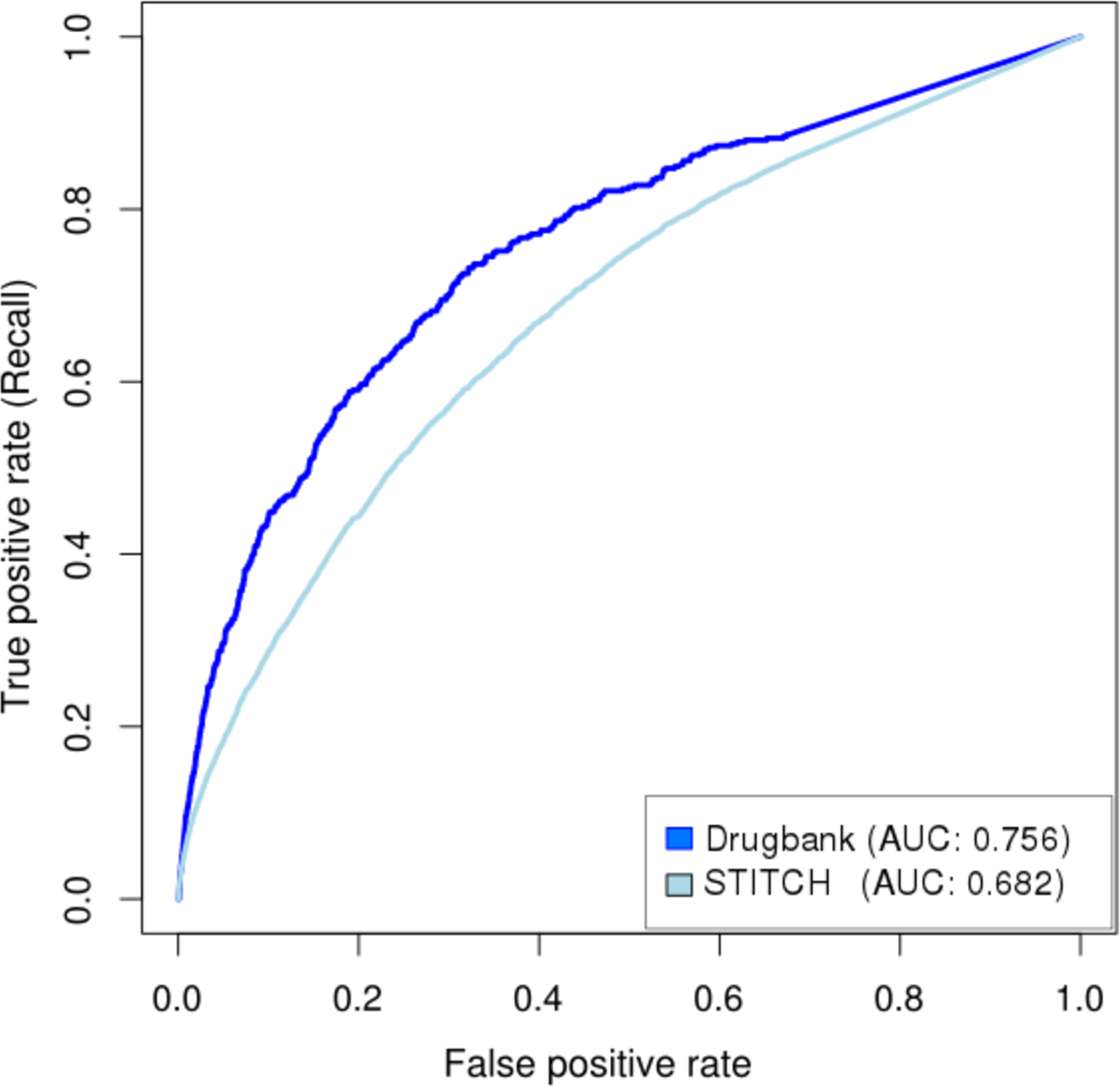

Supplement: S5 Fig — The performance of the method on the benchmark sets that Hoehndorf and collaborators gathered from STITCH (human) (drugeffect-data/positive/stitch-human-targets-0.7.txt) is shown in light blue and from DrugBank (drugeffect-data/positive/drugbank-targets.txt) is depicted in darker blue. (TIF) [file pcbi.1005111.s013.tif]

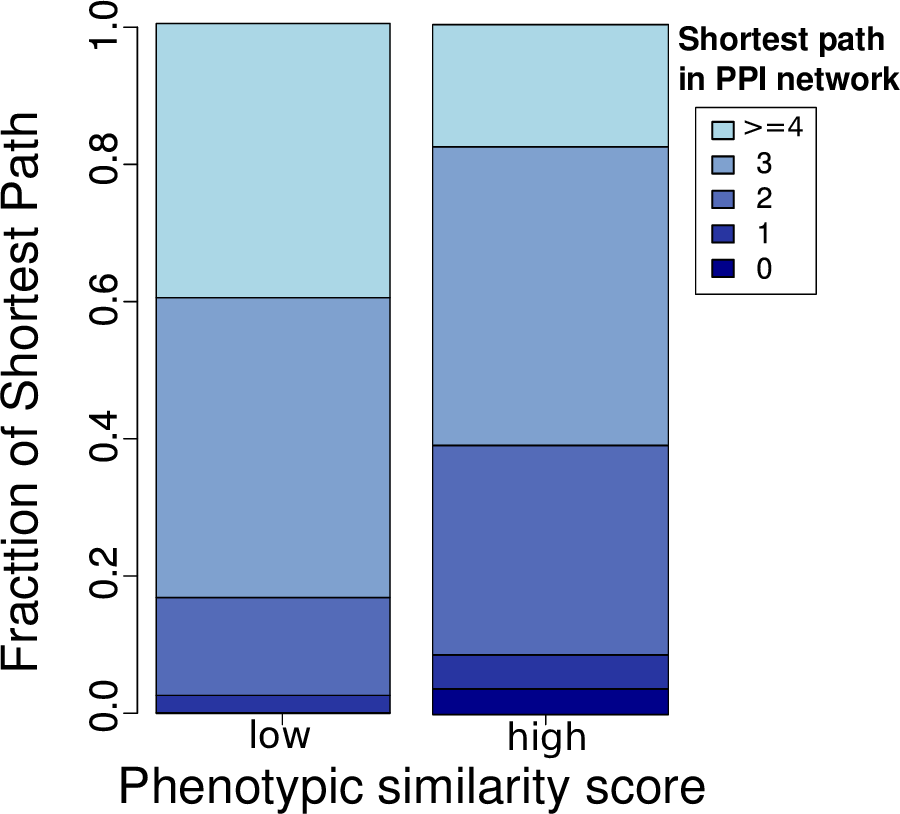

Supplement: S6 Fig — Phenotypically similar drug-gene pairs tend to share close molecular mechanisms, as drug-gene pairs involving a drug target that interacts directly (shortest path distance 0) or through a common protein (shortest path distances 1 or 2) with the protein coded by the human orthologue of the mouse gene are significantly enriched in high semantically similar pairs (P-value = 3.41E-62, Wilcoxon test). (TIF) [file pcbi.1005111.s014.tif]

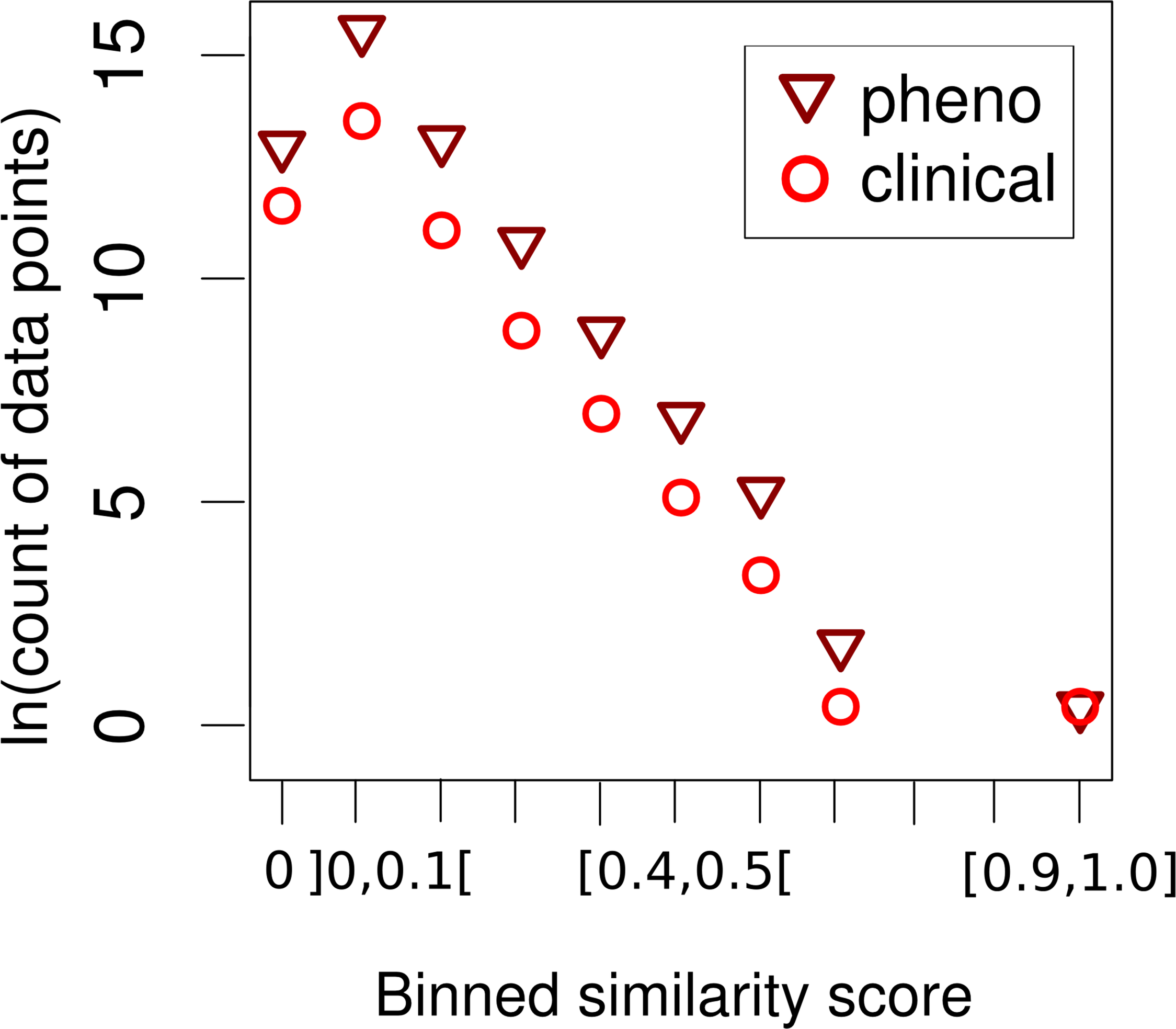

Supplement: S7 Fig — We binned the drug-gene pairs according to their phenotypic similarity and calculated the natural logarithm of the number of drug-gene pairs in the benchmark set of PharmGKB per bin. (TIF) [file pcbi.1005111.s015.tif]

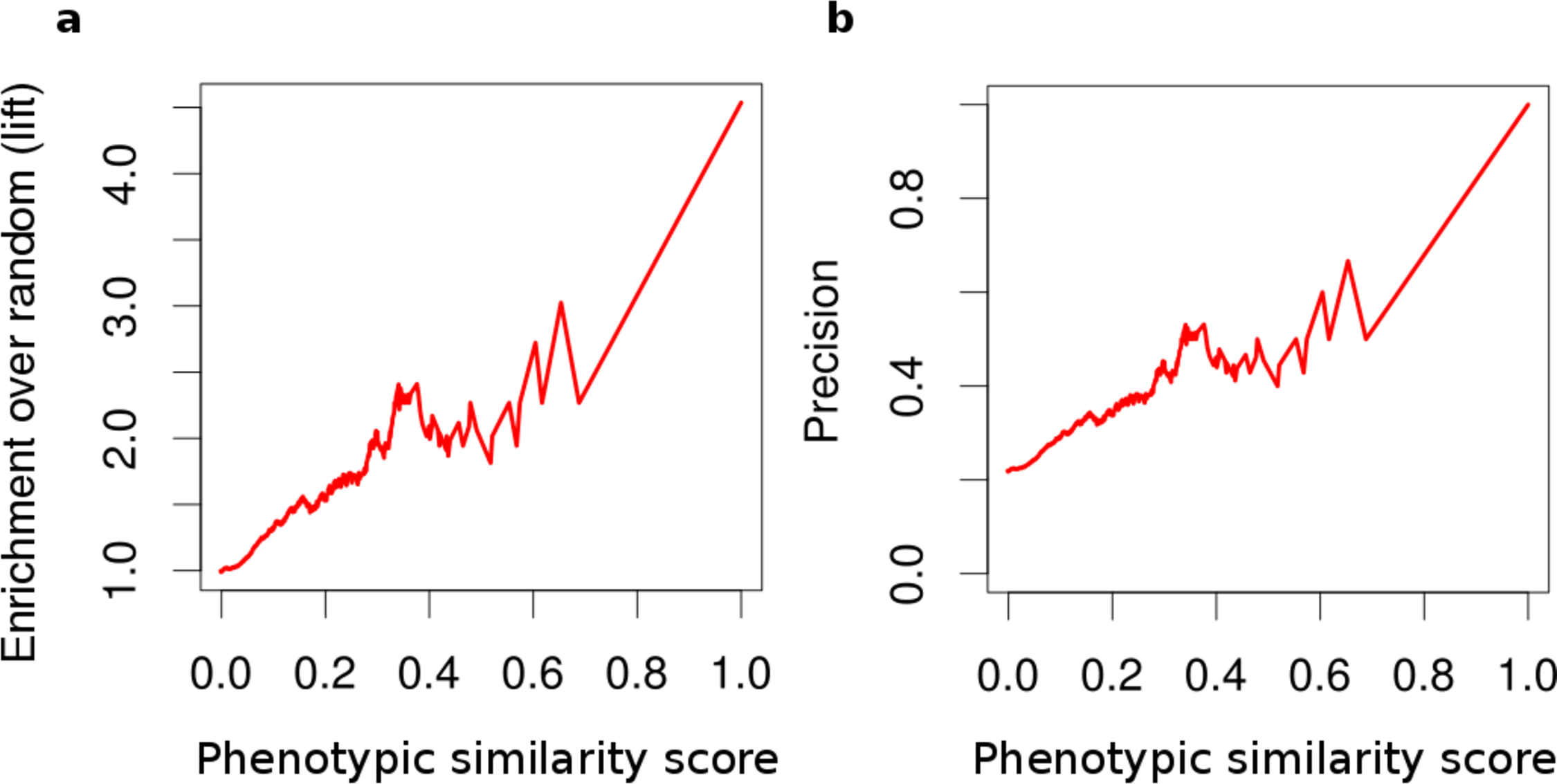

Supplement: S8 Fig — The intended targets of the ToxCast assays were mapped to our set of genes and the compounds to our drug data set. The precision and lift of the resulting 14,238 drug-gene pairs showed the strong tendency of phenotypically similar drug-gene pairs to be a hit in assays of the ToxCast project. (TIF) [file pcbi.1005111.s016.tif]

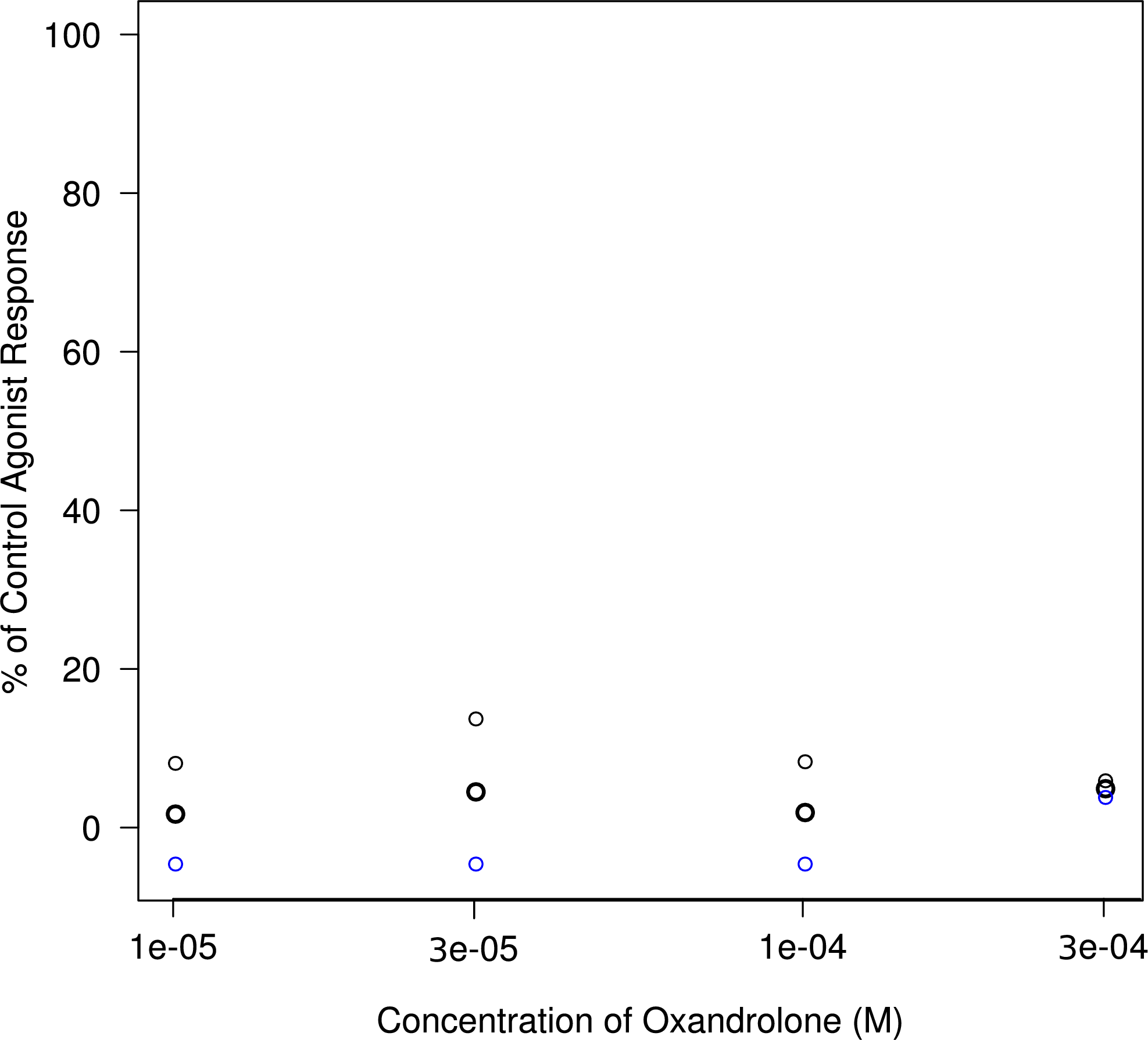

Supplement: S9 Fig — We tested oxandrolone in HEK-293 cells expressing PKR2 in the absence of PK2, the known ligand of the receptor. We could not detect activity of oxandrolone in this assay, even at the highest concentrations tested. This result demonstrates the specificity of the action of oxandrolone on PKR2 signaling, thereby excluding the possibility of interference through a completely different route. (TIF) [file pcbi.1005111.s017.tif]
