# Supplementary material for: A Novel Drug-Mouse Phenotypic Similarity Method Detects Molecular Determinants of Drug Effects
Source: PLoS Comput Biol. 2016 Sep 27;12(9):e1005111. doi: 10.1371/journal.pcbi.1005111 (PMC5038975; doi:10.1371/journal.pcbi.1005111)
Supplement: S1 Text — (DOC) [file pcbi.1005111.s018.doc]

Supplemental Information

**Shortest path in a protein-protein interaction (PPI) network to known drug target**

We investigated if the targets of a drug that is phenotypically similar to a mouse gene are close to the proteins encoded by this mouse gene in a protein-protein interaction (PPI) network. More specifically, we calculated the shortest distance of all direct targets of a drug to the gene product of the human orthologue of the mouse gene in a PPI network for every drug-mouse gene pair (see also supplemental experimental procedures section “Shortest path in a protein-protein interaction (PPI) network to known drug target” for details). We found that high scoring drug-gene pairs tend to share close molecular mechanisms. In particular, we detected that drug-gene pairs involving a drug target (shortest path distances 0) that interacts directly or through a common protein (shortest path distances 1 or 2) with the protein coded by the human orthologue of the mouse gene are significantly enriched in high semantically similar pairs (P-value=3.41E-62, Wilcoxon test) (Figure S6). The pair *CYP19A1* - *dutasteride* illustrates an indirect relationship with a close functional connection between the gene and the drug target. In this pair, both the target of *dutasteride,* steroid-5-alpha-reductase, and the protein encoded by the gene *CYP19A1* are members of the steroid hormone biosynthesis pathway.
